# *foxg1a* is required for hair cell development and regeneration in the zebrafish lateral line

**DOI:** 10.1242/bio.060580

**Published:** 2024-09-20

**Authors:** Jon M. Bell, Emily M. Turner, Cole Biesemeyer, Madison M. Vanderbeck, Roe Hendricks, Hillary F. McGraw

**Affiliations:** ^1^ University of Missouri Kansas City, School of Science and Engineering, Division of Biological and Biomedical Systems, Kansas City, MO 64110, USA; ^2^ Research Organisms, Stowers Institute for Medical Research, Kansas City, MO 64110, USA

**Keywords:** Zebrafish, *foxg1a*, Hair cells, Proliferation, Development

## Abstract

Mechanosensory hair cells located in the inner ear mediate the sensations of hearing and balance. If damaged, mammalian inner ear hair cells are unable to regenerate, resulting in permanent sensory deficits. Aquatic vertebrates like zebrafish (*Danio rerio*) have a specialized class of mechanosensory hair cells found in the lateral line system, allowing them to sense changes in water current. Unlike mammalian inner ear hair cells, lateral line hair cells can robustly regenerate following damage. In mammals, the transcription factor Foxg1 functions to promote normal development of the inner ear. Foxg1a is expressed in lateral line sensory organs in zebrafish larvae, but its function during lateral line development and regeneration has not been investigated. Our study demonstrates that mutation of *foxg1a* results in slower posterior lateral line primordium migration and delayed neuromast formation. In developing and regenerating neuromasts, we find that loss of Foxg1a function results in reduced hair cell numbers, as well as decreased proliferation of neuromast cells. Foxg1a specifically regulates the development and regeneration of Islet1-labeled hair cells. These data suggest that Foxg1 may be a valuable target for investigation of clinical hair cell regeneration.

## Teaser

**Summary:** Our work demonstrates a role for Foxg1a in developing and regenerating mechanosensory hair cells.

## INTRODUCTION

Auditory perception and balance depend on specialized mechanosensory hair cells in the inner ear (Caprara and Peng, 2022; Fettiplace, 2017; Thomas et al., 2015). Damage to these hair cells can occur through multiple mechanisms including genetic mutations, age, prolonged sound exposure, infection, and exposure to ototoxic drugs (Matsui and Cotanche, 2004), resulting in deafness and loss of vestibular function. Mammals are incapable of regenerating lost or damaged hair cells after development, except for small populations of vestibular hair cells (Burns and Stone, 2017). These injuries lead to permanent sensory-motor disability (Matsui and Cotanche, 2004; Roberson and Rubel, 1994). The World Health Organization estimates that as of 2018 hearing loss was the fourth leading cause of disability in humans with more than 466 million individuals diagnosed (World Health Organization, 2018). In turn, the cost of untreated hearing loss reported by the World Health Organization in 2017 was estimated to be greater than 750 billion dollars globally (World Health Organization, 2017). Thus, research into the development of these mechanosensory hair cells and surrounding tissue is an important field for human health and economic stability.

Contrary to their mammalian counterparts, non-mammalian vertebrates are capable of regenerating functional hair cells during development and throughout adulthood (Brignull et al., 2009; Ghysen and Dambly-Chaudiere, 2007; Kniss et al., 2016; Pinto-Teixeira et al., 2015). In addition to inner ear hair cells, zebrafish, as well as other aquatic vertebrates, have a lateral line mechanosensory system that uses hair cells to sense water motion (Brignull et al., 2009; Coombs et al., 2014). Zebrafish lateral line hair cells show developmental, morphological, and genetic conservation with mammalian inner ear hair cells making them an attractive model for studying human development and disease (Nicolson, 2005). The zebrafish lateral line mechanosensory system lends itself particularly well to experimentation due to its superficial localization on the surface of the body, allowing easy visualization and manipulation. The lateral line system is made up of small sensory organs, called neuromasts, arrayed on the surface of the fish. These neuromasts contain mechanosensory hair cells and surrounding support cells (Thomas et al., 2015). Many key molecular pathways, such as FGF, Notch, and Wnt are critical for the development of inner ear and zebrafish lateral line hair cells (Baek et al., 2022; Jiang et al., 2014; Lush et al., 2019). Support cells help form the surrounding tissue in which hair cells reside, provide trophic support for innervating neurons, and act as progenitors for hair cell regeneration (Cruz et al., 2015; Thomas et al., 2015). Support cells' ability to act as progenitors is regulated by FGF, Notch, and Wnt signaling (Lush et al., 2019; Lush and Piotrowski, 2014; Megerson et al., 2024). Zebrafish lateral line hair cells show dose-dependent damage in response to ototoxic drugs, as is also observed in mammalian models and human patients (Brignull et al., 2009; Namdaran et al., 2012). Taken together, the conservation between mammalian hair cells and zebrafish lateral line hair cells makes them a significant tool to investigate disease and damage as well as possible therapeutic interventions.

Forkhead box G1 (Foxg1) is a member of a large family of transcription factors that regulate multiple cellular processes including proliferation, differentiation, and survival (Clark et al., 1993). Foxg1 has been implicated during development to increase proliferation of progenitor cells in multiple tissues including the inner ear tissue (Ding et al., 2020; Hwang et al., 2009; Wong et al., 2019). Recent work shows that Foxg1 is involved in hair cell development and homeostasis in mammalian models (He et al., 2020; Hwang et al., 2009; Zhang et al., 2020). The loss of Foxg1 function results in morphological deformities of the cochlea and sensory cristae, as well as altered hair cell polarity and total hair cell numbers (Hwang et al., 2009; Pauley et al., 2006; Zhang et al., 2020). Foxg1 is also implicated in age related hair cell homeostasis through regulation of reactive oxygen species and autophagy (He et al., 2020). Foxg1 interacts directly with critical hair cell development pathways such as canonical Wnt, FGF, and Notch signaling (Akol et al., 2022; Ding et al., 2020). The possible functions of Foxg1 have not been studied in the developing zebrafish lateral line or in the context of hair cell regeneration. We seek to uncover the function of Foxg1 in the development and regeneration of the zebrafish lateral line.

Our current study investigates the function of Foxg1a using the *foxg1a^a266^* (Thyme et al., 2019) mutant line to examine development and regeneration of hair cells and support cells in the zebrafish posterior lateral line (pLL). We show that loss of Foxg1a function results in slower pLL primordium (pLLP) migration during the development of the pLL and delayed neuromast formation. We found that significantly fewer hair cells form in the nascent pLL and there is a reduction in proliferating cells in the developing neuromasts. Following regeneration, we found a significant reduction in proliferating support cells, hair cells numbers, and α-Isl1-positive cells in *foxg1a^a266^* mutant neuromasts. This work suggests that Foxg1a functions in the neuromast to enable appropriate proliferation and differentiation of cells in the zebrafish lateral line. Understanding the role of Foxg1a in hair cell biology may provide potential directions for future interventions in human hearing loss.

## RESULTS

### *foxg1a* is expressed in the developing and regenerating posterior lateral line

**Fig. 1.**
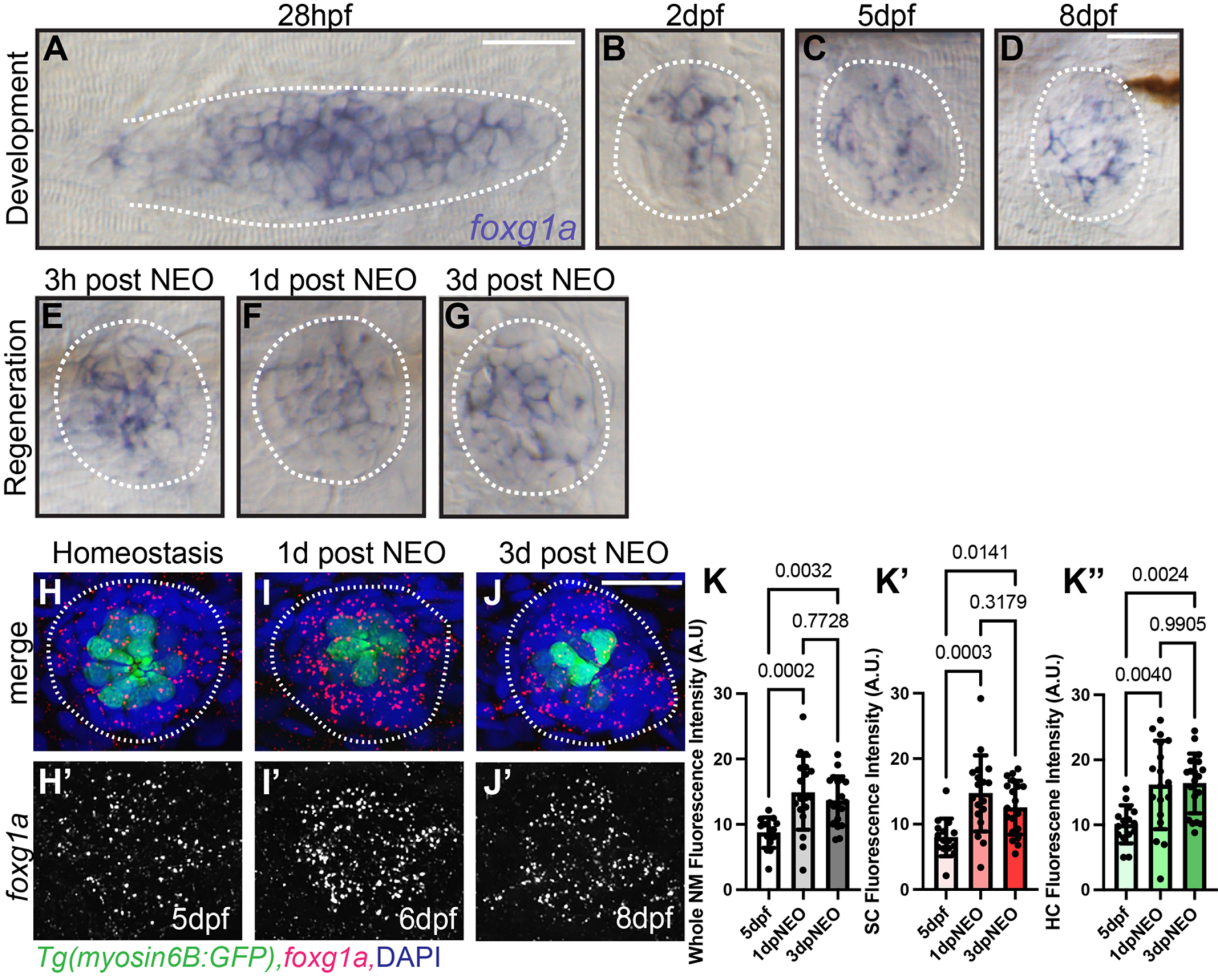
**RNA *in situ* hybridization shows *foxg1a* expression in developing and regenerating posterior lateral line tissue.** (A-D) Wholemount RNA *in situ* hybridization of *foxg1a* in wild-type zebrafish posterior lateral line primordium at 28 hpf (A), and in neuromasts at 2 dpf (B), 5 dpf (C), and 8 dpf (D). (F-G) Wholemount RNA *in situ* hybridization of *foxg1a* in wild-type zebrafish neuromasts during regeneration following neomycin (NEO) exposure at 5 dpf. (E) 3 h post-NEO, (F) 1-day post-NEO, and (G) 3 days post-NEO. (H-J′) Confocal projections of wild-type neuromasts showing hair cells labeled with *Tg(myo6:GFP)* (green), *foxg1a* expression with HCR fluorescent *in situ* hybridization (red) and nuclei labeled with DAPI (blue) at 5 dpf (H), 1 day-post NEO exposure (I), and 3 days-post NEO (J). Quantification of HCR *foxg1a* fluorescence intensity in arbitrary units (A.U.) in the whole neuromasts (K), hair cells (hc; K′), and support cells (sc; K″). *n*=15 NMs (nine larvae) 5 dpf, *n*=16 NMs (nine larvae) 1 day-post NEO, and *n*=18 NMs (nine larvae) 3 days-post NEO). All data presented at mean±s.d. Kruskal–Wallis test with Dunn's multiple comparisons. Scale bars: 20 µm.

Foxg1 function in the development of mammalian inner ear led us to investigate its possible role in the zebrafish lateral line (Ding et al., 2020; Pauley et al., 2006). Work by others has suggested that *foxg1a* is expressed in lateral line neuromasts but as of yet, no functional role has been elucidated (Baek et al., 2022; Lush et al., 2019). We first sought to determine if *foxg1a* is expressed in the developing lateral line. Using wholemount RNA *in situ* hybridization (WISH) in wild-type embryos we show *foxg1a* is expressed in the migrating pLLP at 28 h post fertilization (hpf) (Fig. 1A; Fig. S1A). The migrating pLLP deposits clusters of cells in its wake that will continue to proliferate and differentiate to give rise to neuromasts containing mechanosensory hair cells and surrounding support cells (Brignull et al., 2009; Ma and Raible, 2009; Thomas et al., 2015). We see expression of *foxg1a* in newly deposited wild-type zebrafish neuromasts at 2 days post fertilization (dpf) (Fig. 1B; Fig. S1B) and in maturing neuromasts at 5 dpf (Fig. 1C; Fig. S1C) and 8 dpf (Fig. 1D; Fig. S1D). *foxg1a* expression is maintained in the regenerating neuromast at 3 h post hair cell ablation with the ototoxic aminoglycoside antibiotic neomycin (Fig. 1E; NEO), 1 day-post NEO exposure (Fig. 1F; 1 day-post NEO), and after regeneration is complete at 3 days-post NEO exposure (Fig. 1G; 3 days-post NEO). The continued expression of *foxg1a* in the neuromast during regeneration was also reported by single-cell RNA-sequencing (Baek et al., 2022). We next sought to analyze expression levels and cellular localization of *foxg1a* during homeostasis and regeneration. To do so we used hybridization chain reaction fluorescence *in situ* hybridization (HCR FISH) in wild-type larvae. We observed expression throughout the neuromast during homeostasis, and during regeneration at 1 day-post NEO and 3 days-post NEO (Fig. 1H-J′). We further characterized expression by comparing fluorescent intensity of the whole neuromast, hair cells, and non-hair cell support cells during homeostasis and regeneration. A significant increase in *foxg1a* expression is observed following hair cell ablation in the whole neuromast (Fig. 1K), hair cells (Fig. 1K′), and surrounding support cells (Fig. 1K″) 1 day-post NEO and 3 days-post NEO when compared to 5 dpf non-ablated larvae. Although we find increased *foxg1a* expression during regeneration, we do not find a bias toward specific cell types within the neuromast; *foxg1a* is expressed in hair cells and surrounding support cells at similar levels (Fig. 1K-K″). These data demonstrate that *foxg1a* expression occurs within the migrating pLL primordium and carries on through maturation of LL neuromasts with increased levels of expression during regeneration.

### Foxg1a regulates posterior lateral line primordium migration and neuromast development

**Fig. 2.**
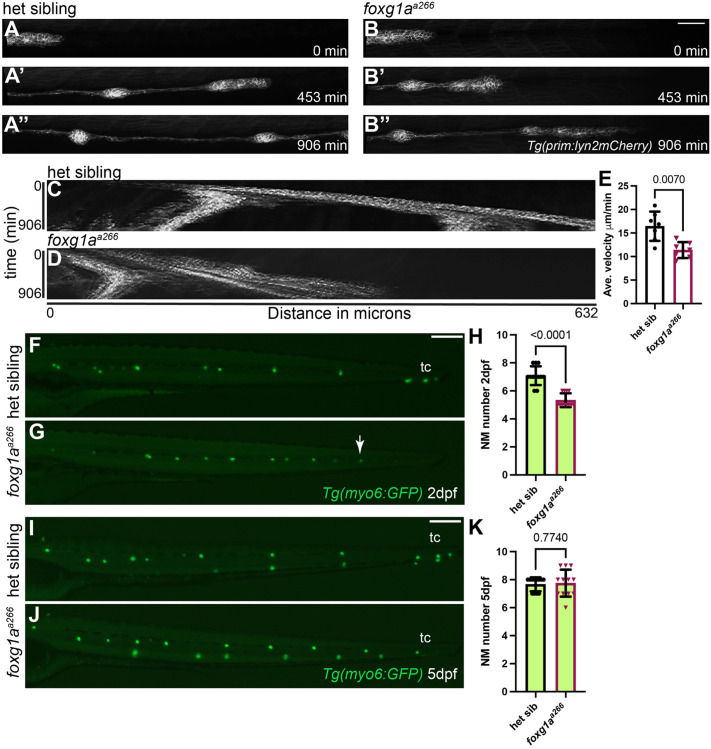
**Loss of Foxg1a results in slower posterior lateral line primordium migration and delayed neuromast formation.** (A-B″) Confocal projections of time lapse video of posterior lateral line migration primordium at 0, 453, and 906 min in heterozygous (A-A″) and *foxg1a^a266^* mutant embryos (B-B″). Scale bar: 100 µm. (C,D) Kymograph of time lapse video of posterior lateral line migration in heterozygous (C) and *foxg1a^a266^* mutant embryos (D). (E) Quantification of average primordium velocity during migration, *n*=7 embryos per condition. (F,G) Live images of *Tg(myo6:GFP*)-labeled neuromasts in heterozygous (F) and *foxg1a^a266^* mutant embryos (G) at 2 dpf, white arrow indicates location of truncated primordium migration and terminal cluster neuromasts at the end of the tail are indicated by tc. (H) Quantification of NM number at 2 dpf, *n*=12 embryos per condition. (I,J) Live images of *Tg(myo6:GFP*)-labeled neuromasts at 5 dpf in heterozygous (I) and *foxg1a^a266^* mutant larvae (J). (K) Quantification of NM number at 5 dpf, *n*=12 larvae per condition. Data presented as mean±s.d., Mann–Whitney *U*-test. Scale bars: 20 µm.

As we observed expression of *foxg1a* in the developing and regenerating lateral line, we sought to determine if it plays a functional role as well. During embryonic development, the zebrafish pLL follows a well characterized and stereotypical developmental pattern that involves the collective migration of the pLLP cells and deposition of proto-neuromasts along the trunk (Ma and Raible, 2009; Thomas et al., 2015). Mutations affecting pLL development result in phenotypes including truncated lateral line formation, supernumerary neuromasts, loss of neuromasts, altered hair cell numbers, and changes in neuromast size (Lush and Piotrowski, 2014; Thomas et al., 2015). The *foxg1a^a266^* mutant allele is predicted to be a null mutation with a large deletion overlapping the Forkhead domain in the single exon of *foxg1a* (Thyme et al., 2019). Neuromasts of the zebrafish pLL form after being deposited in the wake of the migrating pLLP between ∼22 hpf and 48 hpf. Live time-lapse imaging of heterozygous and homozygous *foxg1a^a266^* embryos expressing *Tg(prim:lyn2mCherry)*, a transgene that labels the cell membranes of the migrating primordium and neuromasts, as well as other sensory tissues (Wang et al., 2018), shows pLLP migration velocity between 33-48 hpf is significantly slower in mutants as compared to controls (Fig. 2A-E; Movie 1). We confirmed this reduced velocity was not due to a reduction in the total number of primordia cells in *foxg1a^a266^* larvae (Fig. S2A,A″,B,B″,C′), total area of the pLLP (Fig. S2C″), or a reduction of cellular proliferation in the primordia as observed by BrdU, a thymidine analog, incorporation into newly synthesized DNA during migration (Fig. S2A,A′,B,B′,C). At 2 dpf we found there was a small, but significant reduction in the number of deposited neuromasts in *foxg1a^a266^* mutants as compared to heterozygous controls (Fig. 2F-H). There was no significant change in somite number, embryo length, or eye circumference at 2 dpf, indicating that the slower pLLP migration appears specific to pLL development as opposed to overall developmental delay (Fig. S3A-B′,C,D,E). However, this reduction was recovered by 5 dpf (Fig. 2I-K). These data demonstrate Foxg1a function plays a role in the timing of pLL development during primordium migration and neuromast deposition.

### Hair cell numbers are reduced in *foxg1a^a266^* mutant neuromasts during development and regeneration

**Fig. 3.**
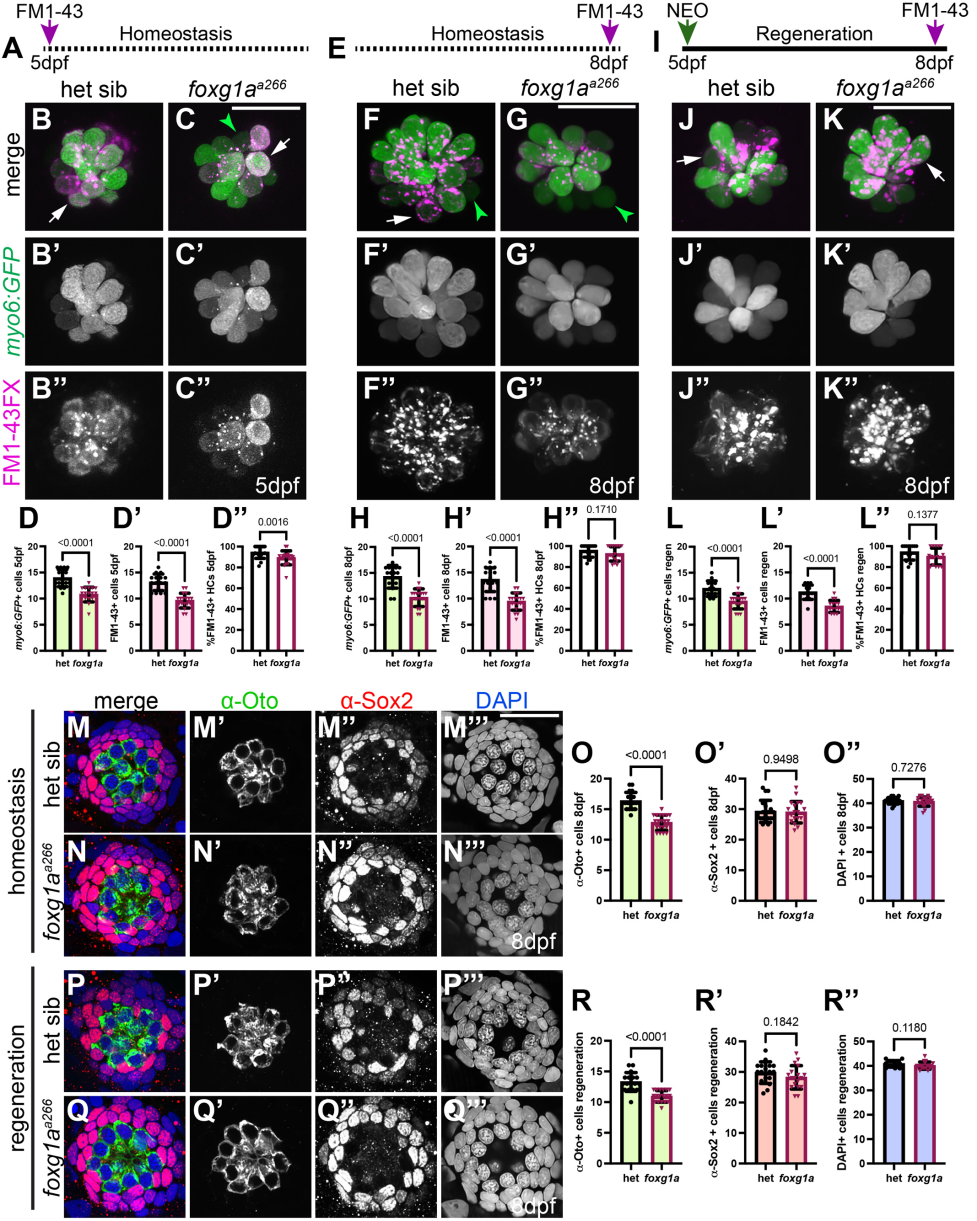
**Loss of Foxg1a function reduces hair cell development and regeneration.** (A) Timeline of FM1-43FX exposure and live imaging at 5 dpf. (B-C″) Live confocal projections of *Tg(myo6:GFP)-*labeled (green) and FM1-43FX-labeled (magenta) neuromasts in heterozygous (B-B″) and *foxg1a^a266^* mutant larvae (C-C″) at 5 dpf, white arrows indicate co-labeling of *myo6:GFP* and FM1-43FX and green arrowheads indicate only *myo6:GFP* labeling. (D-D″) Quantification of *myo6(GFP*)+ hair cells (D), FM1-43FX+ hair cells (D′), and the percentage of FM1-43FX+ hair cells (D″) at 5 dpf. *n*=17 neuromasts (11 larvae) heterozygous sibling and *n*=19 neuromasts (ten larvae) *foxg1a^a266^* mutants. (E) Timeline of FM1-43FX exposure and live imaging at 8 dpf. (F-G″) Live confocal projections of *Tg(myo6:GFP)-*labeled (green) and FM1-43FX-labeled (magenta) neuromasts in heterozygous (F-F″) and *foxg1a^a266^* mutant larvae (F-G″) at 8 dpf. (H-H″) Quantification of *myo6(GFP*)+ hair cells (H), FM1-43FX+ hair cells (H′), and the percentage of FM1-43FX+ hair cells (H″) at 8 dpf. *n*=16 neuromasts (eight larvae) heterozygous sibling and *n*=17 neuromasts (nine larvae) *foxg1a^a266^* mutants. (I) Time line of NEO-exposure, regeneration, and FM1-43 labeling. (J-K″) Live confocal projections of *Tg(myo6:GFP)-*labeled (green) and FM1-43FX-labeled (magenta) neuromasts in heterozygous (J-J″) and *foxg1a^a266^* mutant larvae (K-K″) following regeneration at 8 dpf. (L-L″) Quantification of Tg(*myo6:GFP*)+ hair cells (L), FM1-43FX+ hair cells (L′), and the percentage of FM1-43FX+ hair cells (L″) following regeneration. *n*=14 neuromasts (seven larvae) heterozygous sibling and *n*=15 neuromasts (eight larvae) *foxg1a^a266^* mutants. (M-N′″) Confocal projections of 8 dpf larvae showing hair cells labeled with α-Oto antibody (green), support cells labeled with α-Sox-2 antibody (red), and nuclei labeled with DAPI in heterozygous sibling (M-M′″) and *foxg1a^a266^* (N-N′″) neuromasts. (O-O″) Quantification of α-Oto+, α-Sox-2 +, and DAPI+ cells. *n*=19 neuromasts (ten larvae) heterozygous sibling and *n*=20 neuromasts (ten larvae) *foxg1a^a266^* mutants. (P-Q′″) Confocal projects of 8dpf larvae following NEO-exposure and regeneration showing hair cells labeled with α-Oto antibody (green), support cells labeled with α-Sox-2 antibody (red), and nuclei labeled with DAPI in heterozygous sibling (P-P′″) and *foxg1a^a266^* (Q-Q′″) neuromasts. (R-R″) Quantification of regenerated α-Oto+, α-Sox-2 +, and DAPI+ cells. *n*=17 neuromasts (nine larvae) heterozygous sibling and *n*=18 neuromasts (nine larvae) *foxg1a^a266^* mutants. Data presented as mean ±s.d., Mann–Whitney *U*-test. Scale bars: 20 µm.

Cells in the maturing zebrafish neuromasts form mechanosensory hair cells and surrounding support cells (Coombs et al., 2014; Ghysen and Dambly-Chaudiere, 2007; Thomas et al., 2015). As Foxg1a function seems necessary for early lateral line development and is expressed in maturing neuromasts, we next asked if it was necessary for hair cell development. During pLLP migration, the first hair cells will start to form before being deposited with developing neuromasts (Itoh and Chitnis, 2001). Using the *Tg(mysoin6b:GFP)^w186^ (myo6:GFP)* transgenic zebrafish, which will label hair cells, we are able to visualize and quantify hair cell numbers. At 28 hpf we can see the formation of early hair cells in the trailing pLLP with no significant difference in quantity when comparing *foxg1a^a266^* embryos to heterozygous controls (Fig. S2D,D′,F,F,G). Looking at later developmental time points we find on average significantly fewer hair cells in *foxg1a^a266^* mutant pLL neuromasts compared to heterozygous siblings (Fig. 3A,B-D′) at 5 dpf when the majority of hair cells in the developing neuromast have matured. The reduction in hair cells is also observed at 8 dpf, suggesting it is not developmental delay (Fig. 3E,F-H″). To determine if hair cells are being lost to apoptosis, we conducted TUNEL assays at 4 dpf and 8 dpf and found no significant increase in cell death (Fig. S4A-H). We quantified the population of hair cells with functional mechanoelectrical transduction (MET) channels using the fixable fluorescent vital dye FM1-43FX, which enters hair cells through MET channels and can give insight into functionality of hair cells (Fettiplace, 2017). We observe there is also a significant reduction in FM1-43FX labeled hair cells in *foxg1a^a266^* larvae as compared to controls at 5 dpf (Fig. 3B,B″,C,C″,D′) and that the percent of FM1-43FX-labeled hair cells is significantly reduced in mutant larvae (Fig. 3D″). Looking later in development we conducted this same experiment at 8 dpf and observed the same significant reduction in *myo6:GFP* and FM1-43FX-labeled hair cells in *foxg1a^a266^* larvae as compared to heterozygous siblings (Fig. 3F,F″,G,G″,H′), however, the percent of FM1-43FX labeled cells is no longer reduced in the 8 dpf mutant population (Fig. 3H″). These data demonstrate that Foxg1a function is necessary for the appropriate number of hair cells to develop in the zebrafish lateral line.

Many of the molecular and cellular mechanisms that drive hair cell development in the zebrafish lateral line are also active during regeneration (Cruz et al., 2015; Kniss et al., 2016; Pinto-Teixeira et al., 2015). For that reason, we asked if Foxg1a function is required for regeneration of hair cells. We used NEO-exposure to ablate hair cells in 5 dpf zebrafish larvae when the majority of hair cells are mature (Harris et al., 2003) and assessed regeneration at 8 dpf (Fig. 3I). At 3 days-post NEO we again used *myo6:GFP* to label total hair cell numbers and found that *foxg1a^a266^* larvae had fewer average hair cells per neuromast as compared to heterozygous siblings (Fig. 3J,J′,K,K′,L). When we use FM1-43FX to measure hair cells with functional MET channels 3 days-post NEO, we find a significant reduction in average hair cells per neuromast in *foxg1a^a266^* larvae as compared to controls (Fig. 3J,J″,K,K″,L′), which recapitulates observations of homeostatic conditions at 5 dpf and 8 dpf. The percent of FM1-43FX labeled *foxg1a^a266^* hair cells is not significantly different 3 days-post NEO (Fig. 3L″). A TUNEL assay at 18 h-post NEO, when proliferation is at its peak during regeneration (Ma et al., 2008), showed no significant difference in labeling between heterozygous sibling and *foxg1a^a266^* mutants (Fig. S4I-K), suggesting cell death is not the major cause of the reduction of regenerated hair cells in *foxg1a^a266^* mutant larvae. These data indicate that Foxg1a function is necessary for the development and regeneration of neuromast hair cells, and that that these hair cells have functional MET channels. We must note that these experiments do not fully confirm functional transduction of signals between hair cells and innervating neurons. Mutations affecting hair cell and neuromast development can also cause disruptions in innervation and hair cell polarity (Kindt and Sheets, 2018; Navajas Acedo et al., 2019). Using *Tg(neuroD:eGFP)* transgenic fish, which labels the axons of innervating neuromast neurons and other tissue (Obholzer et al., 2008), we assessed innervation of *foxg1a^a266^* neuromasts and noted no significant difference when compared to control larvae (Fig. S5A,A″,B,B″,C′). Similarly, we observed no significant difference in hair cell polarity when comparing *foxg1a^a266^* larvae to heterozygous siblings (Fig. S5D-F).

### Total neuromast cell numbers are not reduced in *foxg1a^a266^* mutants

As loss of Foxg1a function results in fewer hair cells, we asked whether there was also a reduction in the non-sensory support cells within the developing neuromast. To look at total support cell populations we used α-Sox2 antibody labeling and noted no significant difference in total support cells when comparing *foxg1a^a266^* mutants to heterozygous siblings at 5 dpf (Fig. 3M,M″,N,N″,O′), though there was still a reduction in hair cells labeled with α-Otoferlin (α-Oto; Fig. 3M,M′,N,N′,O). We also noted there was no change in total DAPI-labeled cells in *foxg1a^a266^* mutants as compared to heterozygous controls (Fig. 3M,M′″,N,N′″,O″). We reasoned there may be a significant difference in support cell populations during regeneration with loss of Foxg1a function. We used NEO exposure to ablate hair cells at 5 dpf then allowed regeneration before fixation and labeling with α-Sox2, and α-Oto to measure any loss of total support cells. We found that at 3 days-post NEO there was no significant decrease in α-Sox2 labeled cells when comparing *foxg1a^a266^* larvae to heterozygous siblings (Fig. 3P,P″,Q,Q″,R′). Similar to observations at 5 dpf during homeostatic analyses, we also saw no significant decrease in total cell numbers as labeled with DAPI (Fig. 3P,P′″,Q,Q′″R″), but still observe the reduced hair cell phenotype when comparing *foxg1a^a266^* mutants to controls (Fig. 3P,P′,Q,Q′,R).

### Proliferation is reduced in *foxg1a^a266^* mutant neuromasts during development

**Fig. 4.**
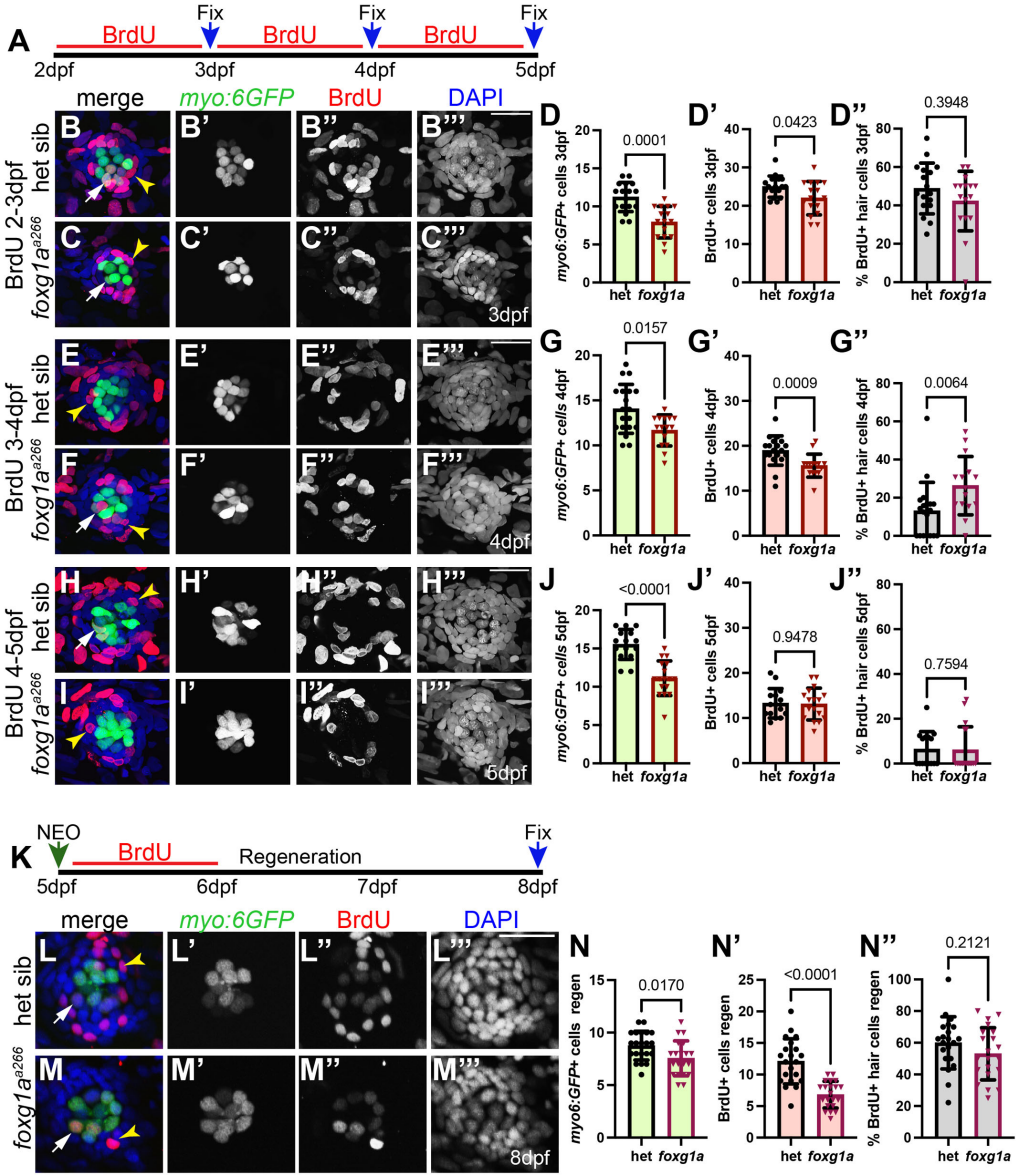
**BrdU incorporation in foxg1a mutants is reduced during neuromast maturation.** (A) Timeline of BrdU incorporation between 2 dpf and 5 dpf. (B-C′″-E,F′″,H-I′″,L-M′″) Confocal projections of heterozygous sibling and *foxg1a^a266^* embryos expressing *Tg(myo6:GFP*) (green) following 24 h windows of BrdU (red) exposure and nuclei labeled with DAPI (blue) between 2-5 dpf. White arrows indicate representative myo6:GPF+ hair cells co-labeled with BrdU. Yellow arrowheads indicate representative neuromast cells labeled with BrdU. (B-B′″) 3 dpf heterozygous sibling and (C-C′″) *foxg1a^a266^* mutant neuromast exposed to BrdU from 2-3 dpf. (D-D″) Quantification of heterozygous sibling and *foxg1a^a266^* hair cells (D), total BrdU incorporation (D′), and percent of BrdU to hair cells (D″) at 3 dpf. *n*=17 neuromasts (nine larvae) heterozygous sibling, *n*=16 neuromasts (eight larvae) *foxg1a^a266^*. (E-F′″) 4 dpf heterozygous sibling (E-E′″) and *foxg1a^a266^* mutant (F-F′″) neuromasts exposed to BrdU from 3-4 dpf. (G-G″) Quantification of heterozygous siblings and *foxg1a^a266^* hair cells (G), total BrdU incorporation (G′), and index of BrdU to hair cells (G″) at 4 dpf. *n*=19 neuromasts (nine larvae) heterozygous sibling, *n*=15 neuromasts (eight larvae) *foxg1a^a266^*. (H-I′″) 5 dpf heterozygous sibling (H-H′″) and *foxg1a^a266^* mutant (I-I″) neuromasts exposed to BrdU from 4-5 dpf. (J-J″) Quantification of heterozygous siblings and *foxg1a^a266^* hair cells (J), total BrdU incorporation (J′), and index of BrdU to hair cells (J″) at 5dpf. *n*=15 neuromasts (eight larvae) heterozygous sibling, *n*=17 neuromasts (eight larvae) *foxg1a^a266^*. (K) Timeline of NEO expose at 5 dpf, followed by 24 h of BrdU incubation and then regeneration through to 8 dpf and fixation. (L-M′″) Confocal projections at 3 days-post NEO treatment at 8 dpf; hair cells and labeled with *Tg(myo6:GFP)* (green), proliferating cells are labeled by BrdU-incorporation (red), and nuclei labeled with DAPI (blue) in heterozygous sibling (L-L′″) and *foxg1a^a266^* mutant (M-M′″) neuromasts. (N-N″) Quantification of hair cells (N), BrdU-labeled cells (N′), and % of BrdU+ hair cells (N″). *n*=21 neuromasts (7 larvae) per condition. All data presented at mean±s.d. Mann–Whitney *U*-tests. Scale bars: 20 µm.

Since we observed a reduction in hair cells with no apparent increase in cell death, we reasoned that the hair cells may not be generated due to effects on cellular proliferation in *foxg1a^a266^* mutant larvae. To investigate this, we used BrdU incorporation in 24-h pulses between 2-5 dpf (Fig. 4A), during the initial maturation of pLL neuromasts. We find significantly fewer cells labeled by BrdU incorporation and fewer hair cells in *foxg1a^a266^* mutant neuromasts as compared to heterozygous controls in larvae exposed to BrdU between 2-3 dpf (Fig. 4B-B″,C,C″,D′) and between 3-4 dpf (Fig. 4E,E″,F,F″,G′). During these same time points we again observe a significant reduction in average hair cell number per neuromast in *foxg1a^a266^* larvae as compared to controls (Fig. 4D,G). At 2-3 dpf the percent of hair cells labeled with BrdU is not significantly different (Fig. 4D″); however, both mutant and control fish pulsed with BrdU from 3-4 dpf demonstrated a reduced percentage of BrdU labeled hair cells compared to the 2-3 dpf (Fig. 4D″,G″). Of the fish pulsed with BrdU 3-4 dpf, a significantly larger percent of BrdU-labeled hair cells was observed when comparing *foxg1a^a266^* embryos to heterozygous siblings (Fig. 4G″). The total number of cells labeled with BrdU incorporation during the 3-4 dpf time point was significantly reduced in the *foxg1a^a266^* embryos as compared to heterozygous siblings (Fig. 4G′), as was observed during the 2-3 dpf time period. We take these data to suggest that loss of Foxg1a function results in fewer hair cells as a consequence of reduced proliferation during neuromast development. To confirm we did not see a recovery or prolonged BrdU incorporation in *foxg1a^a266^* mutants, we pulsed larvae from 4-5 dpf and observed no significant difference in number of or percent of BrdU incorporation in *foxg1a^a266^* larvae as compared to heterozygous siblings while still showing a reduction in hair cells (Fig. 4H-J″).

### Proliferation is reduced during regeneration in *foxg1a^a266^* neuromasts

As loss of Foxg1a function results in reduced proliferation in developing neuromasts, we asked if there is a similar reduction during regeneration. To assess proliferation, we incubated larvae in BrdU for the first 24 h immediately following hair cell ablation with NEO when proliferative regeneration of hair cells is known to occur (Ma et al., 2008). Larvae were then allowed a further 2 days of regeneration before fixation and analysis (Fig. 4K). We observed a significant decrease in BrdU-labeled cells in the *foxg1a^a266^* neuromasts as compared to heterozygous siblings (Fig. 4L,L″,M,M″,N′). We again questioned if there was a delay or recovery of the proliferation phenotype during regeneration. Following NEO-exposure, we pulsed cohorts of larvae with BrdU from 5-6 dpf, 6-7 dpf, or 7-8 dpf (Fig. S6A) then fixed all the larvae at 8 dpf when regeneration was complete. BrdU incorporation showed a significant decrease during the first 24 h pulse (Fig. S6B-C′″,E) demonstrating our initial observations (Fig. 4N′); however, no significant difference was observed in BrdU incorporation of *foxg1a^a266^* larvae as compared to controls during the 6-7 dpf, or the 7-8 dpf (Fig. S6G-O). Though the number of cells observed to have BrdU incorporation was reduced in *foxg1a^a266^* larvae, the percentage of BrdU-labeled hair cells was not significantly different between *foxg1a^a266^* mutant neuromasts and heterozygous controls following regeneration (Fig. 4L,L″,M,M″,N″). We take these data to indicate that loss of Foxg1a function does not impede the proliferative regeneration of hair cells, but it does result in an overall reduction in proliferation and hair cell regeneration.

### Support cell populations are intact in *foxg1a^a266^* mutant neuromasts

**Fig. 5.**
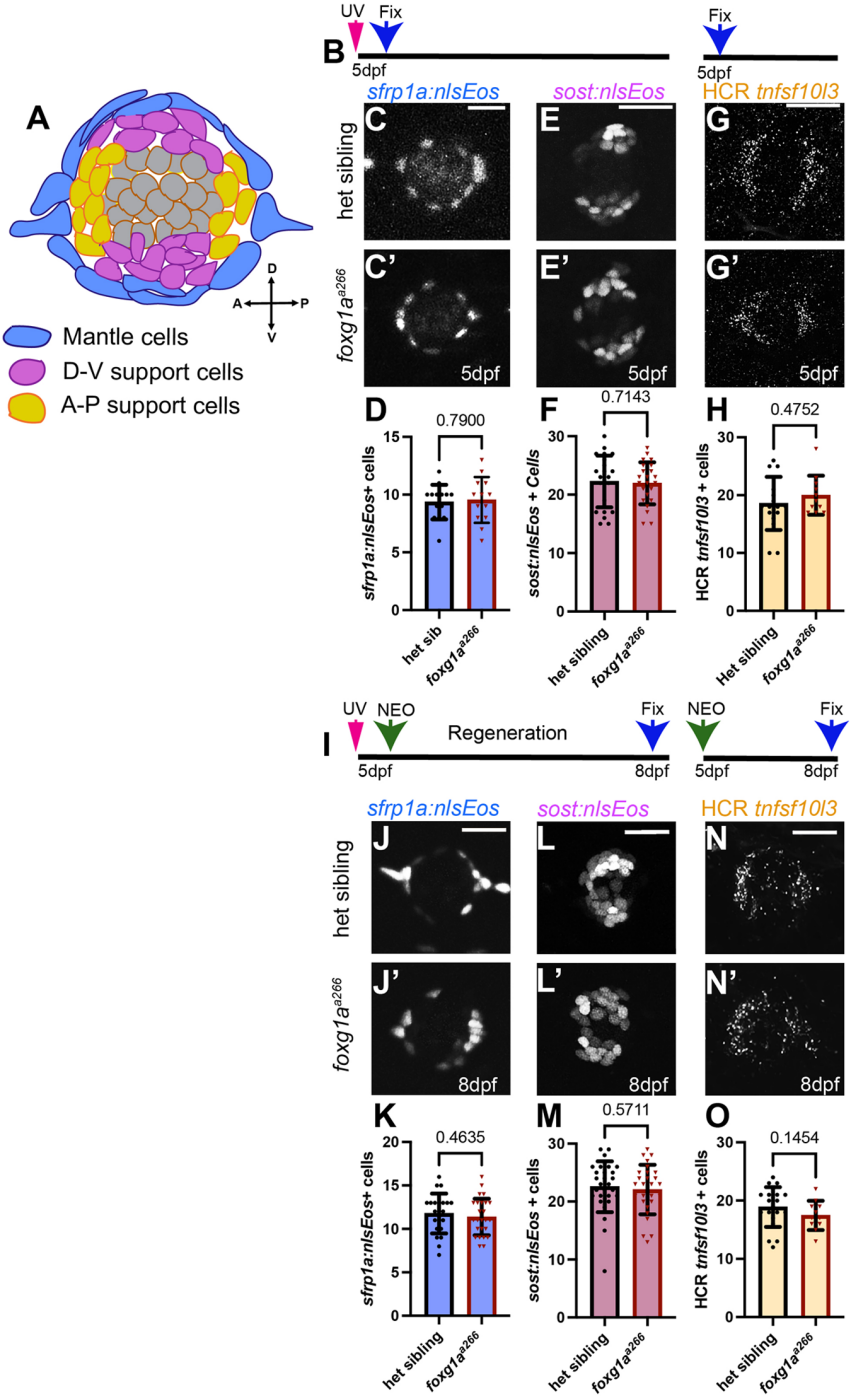
**Support cell populations are unaffected by loss of Foxg1a during development.** (A) Schematic of a neuromast showing peripheral mantle cells (blue), dorsoventral cells (purple), and anterior-posterior cells (yellow). (B) Timeline of UV photo-conversion of nlsEos and fixation of larvae at 5 dpf. (C-C′) Confocal projection of photo-converted *Tg(sfrp1a:nlsEos)-*expressing support cells in 5 dpf heterozygous sibling (C) and *foxg1a^a266^* larvae (C′). (D) Quantification of *Tg(sfrp1a:nlsEos)-*positive dorsoventral support cells. *n*=14 neuromasts (eight larvae) heterozygous sibling, *n*=13 neuromasts (six larvae) *foxg1a^a266^*. (E-E′) Confocal projection of *Tg(sost:nlsEos*)-expressing dorsoventral support cells in 5 dpf heterozygous sibling (E) and *foxg1a^a266^* larvae (E′). (F) Quantification of *Tg(sost:nlsEos)-*positive dorsoventral support cells. *n*=23 neuromasts (eight larvae) per condition. (G-G′) Confocal projections of FISH HCR for *tnfsf10l3* in 5 dpf heterozygous sibling (G) and *foxg1a^a266^* larvae (G′). (H) Quantification of *tnfsf10l3*+ cells. *n*=17 neuromasts (eight larvae) heterozygous, *n*=11 neuromasts in *foxg1a^a266^*. (I) Timeline of photo-conversion and regeneration to 8 dpf after NEO-exposure at 5 dpf. (J-J′) Confocal projection of photo-converted *Tg(sfrp1a:nlsEos)-*expressing support cells in 8 dpf heterozygous sibling (J) and *foxg1a^a266^* larvae (J′). (K) Quantification of *Tg(sfrp1a:nlsEos)-*positive support cells. *n*=22 neuromasts (eight larvae) heterozygous sibling, and *n*=27 neuromasts (ten larvae) *foxg1a^a266^*. (L-L′) Confocal projection of photo-converted *Tg(sost:nlsEos*)-expressing dorsoventral support cells in 8 dpf heterozygous sibling (L) and *foxg1a^a266^* larvae (L′). (M) Quantification of *Tg(sost:nlsEos)-*positive support cells. *n*=28 neuromasts (ten larvae) heterozygous sibling, *n*=27 neuromasts (nine larvae) *foxg1a^a266^*. (N-N′) Confocal projections of HCR fluorescent *in situ* for *tnfsf10l3* in 8 dpf heterozygous sibling (N) and *foxg1a^a266^* larvae (N′). (O) Quantification of *tnfsf10l3*+ cells. *n*=20 neuromasts (ten larvae) heterozygous sibling larvae, and *n*=11 neuromasts in seven *foxg1a^a266^* larvae. All quantification data presented as mean±s.d. Mann–Whitney *U*-test. Scale bars: 20 µm.

During regeneration, differing support cell subpopulations give rise to new hair cells and/or replenish support cells based on location within the zebrafish neuromast (Lush et al., 2019; Thomas and Raible, 2019). Work by others demonstrated that regenerating hair cells arise predominantly from dorsoventral support cells (Romero-Carvajal et al., 2015; Thomas and Raible, 2019) and tend to be located more central to the neuromast as opposed to the periphery (Lush et al., 2019). We asked if there may be a difference in these support cell sub-populations during development and regeneration. These subpopulations are distinguished based on their transcriptomic profile and spatial organization within the neuromast (Fig. 5A) (Lush et al., 2019; Thomas and Raible, 2019). We examined these neuromast support cell sub-populations using *Tg(sfrp1a:nlsEos)^w217^ (sfrp1a:nlsEos)* to label the peripheral mantle cells (Thomas and Raible, 2019) (Fig. 5G-H) and *Tg(sost:nlsEos)^w215^* (*sost:nlsEos*) to label dorsoventral support cells (Fig. 5C-D′) (Thomas and Raible, 2019). These transgenes express nuclear-localized Eos (nlsEos) under the control of the endogenous *sfrp1a* and *sost* promoters (Thomas and Raible, 2019). As a photoconvertible fluorophore Eos will permanently shift from green fluorescent emission to red fluorescent emission when exposed to UV light, allowing for lineage tracing (Wiedenmann et al., 2004). We first quantified the mantle cell population expressing *sfrp1a:nlsEos* and dorsoventral population expressing *sost:nlsEos* at 5 dpf by counting the total photoconverted cells under homeostatic conditions (Fig. 5B). We found no significant difference in the number of labeled cells in *foxg1a^a266^* fish as compared to heterozygous siblings (Fig. 5C-F) when looking at the labeled mantle cells or dorsoventral cells. Using HCR FISH, we quantified the number of cells expressing an anteroposterior support cell marker *tnfsf10l3* at 5 dpf (Fig. 5B) and found no significant difference in the number of labeled cells between *foxg1a^a266^* and heterozygous sibling larvae (Fig. 5G,H). To look at these same cell populations during regeneration we photoconverted *sfrp1a:nlsEos-*positive cells or *sost:nlsEos-*positive cells prior to NEO-exposure, then ablated hair cells with NEO, and traced the fate of *nlsEos*-positive cells following regeneration (Fig. 5I). We found no significant difference in the total number of either *sfrp1a:nlsEos-*labeled cells or *sost:nlsEos*-labeled cells following regeneration when comparing heterozygous and *foxg1a^a266^* larvae (Fig. 5J-M). To assess whether the anteroposterior support cell population was perturbed during regeneration with loss of Foxg1a we again used HCR FISH of *tnfsf10l3* and found no significant difference in the number of cells showing fluorescence in mutant larvae compared to controls (Fig. 5N,O). These data suggest that the mantle cell, dorsoventral, and anteroposterior support cell populations are not sensitive to loss of Foxg1a function.

### mRNA expression patterns are largely unchanged in *foxg1a^a266^* neuromasts

**Fig. 6.**
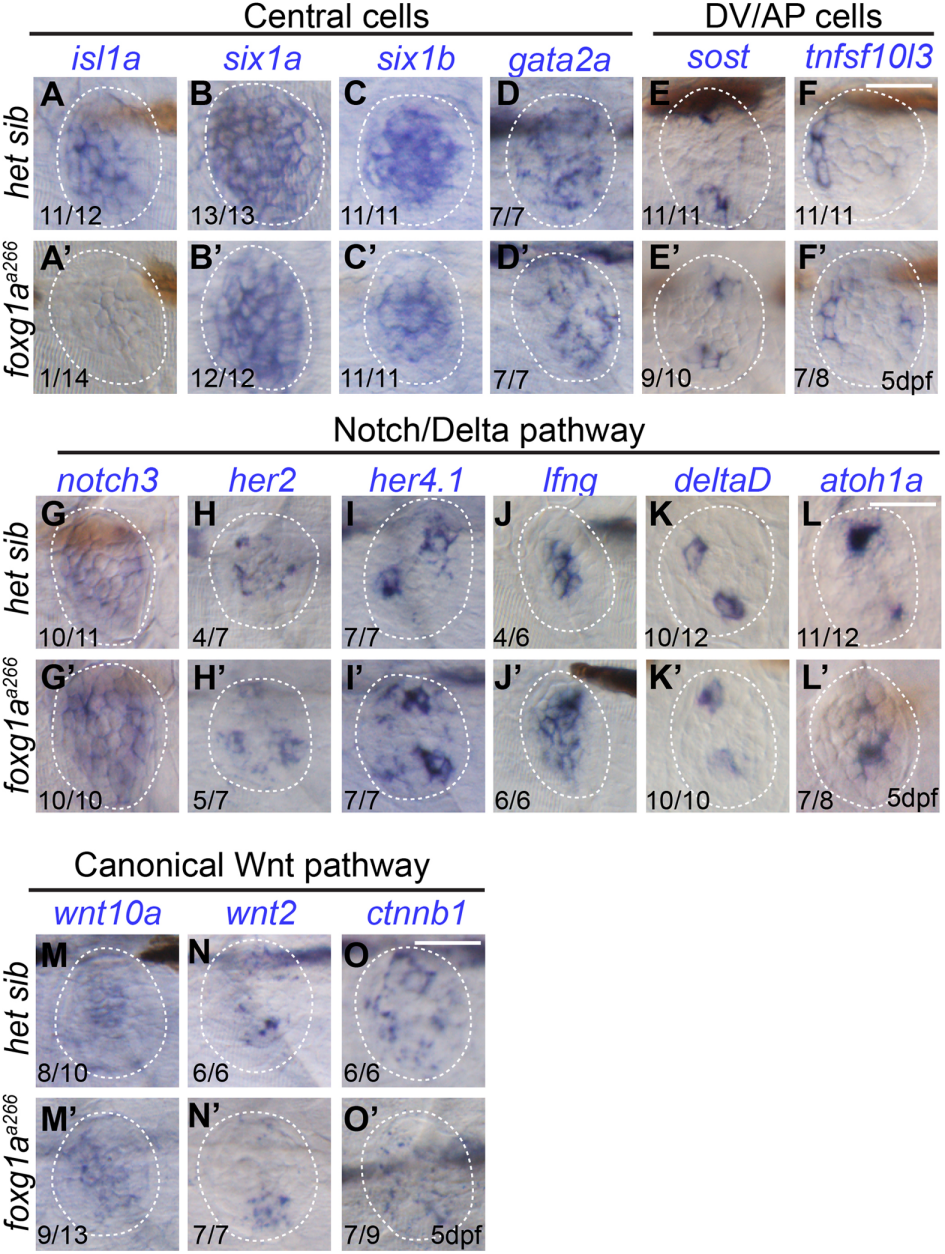
**RNA *in situ* hybridization of NM cell markers.** DIC images of wholemount RNA *in situ* hybridization showing mRNA expression in NMs at 5 dpf in heterozygous sibling (het sib) and *foxg1a^a266^* larvae, lower lefthand numbers indicate the number of larvae with expression in neuromasts over the total number analyzed. Central cell markers: *isl1a* expression in 11/12 het sib (A) and 1/14 *foxg1a^a266^* larvae (A′), *six1a* expression in 13/13 het sib (B) and 12/12 *foxg1a^a266^* larvae (B′), *six1b* expression in 11/11 het sib (C) and 11/11 *foxg1a^a266^* larvae (C′), and *gata2a* expression in 7/7 het sib (D) and 7/7 *foxg1a^a266^* larvae (D′). Dorsoventral and anterior-posterior (DV/AP) cell markers: *sost* expression in 11/11 het sib (E) and 9/10 *foxg1a^a266^* larvae (E′) and *tnfs10l3* expression in 11/11 het sib (F) and 7/8 *fox1ga^a266^* larvae (F′). Expression of Notch/Delta pathways markers: *notch3* expression in 10/11 het sib (G) and 10/10 *foxg1a^a266^* larvae (G′), *her7* expression in 4/7 het sib (H) and 5/7 *foxg1a^a266^* larvae (H′), *her4.1* expression in 7/7 het sib (I) and 7/7 *foxg1a^a266^* larvae (I′), *lfng* expression in 4/6 het sib (J) and 6/6 *foxg1a^a266^* larvae (J′), *deltaD* expression in 10/12 het sib (K) and 10/10 *foxg1a^a266^* larvae (K′), and *atoh1a* expression in 11/12 het sib (L) and 7/8 *foxg1a^a266^* larvae (L′). Canonical Wnt pathway: *wnt10a* expression in 8/10 het sib (M) and 9/13 *foxg1a^a266^* larvae (M′), *wnt2* expression in 6/6 het sib (N) and 7/7 *foxg1a^a266^* larvae (N′), and *ctnnb1* expression in 6/6 het sib (O) and 7/9 *foxg1a^a266^* larvae (O′). Scale bars: 20 µm.

During development, cells deposited in maturing neuromasts will give rise to hair cells, surrounding support cells, and quiescent mantle cells (Brignull et al., 2009; Ma and Raible, 2009). Previous work has demonstrated there are subpopulations of neuromast support cells that show differential function as well as genetic expression (Baek et al., 2022; Lush et al., 2019; Megerson et al., 2024; Thomas and Raible, 2019). Using WISH at 5 dpf, we compared the expression profiles of different genes based on their roles in proliferation and generation of hair cells, cell population localization, and signaling pathways to better understand how loss of Foxg1a function is causing a reduction in hair cells. Based on scRNA-sequencing screens from the Piotrowski and Burgess labs, we investigated four separate genes corresponding to cells central to the neuromast, *isl1a*, *six1a*, *six1b*, and *gata2a* (Jimenez et al., 2022; Lush et al., 2019)*.* At 5 dpf we observe similar patterns of expression for *six1a*, *six1b*, and *gata2a* in *foxg1a^a266^* larvae when compared to controls (Fig. 6B-D′). Surprisingly, though the other central cell markers appeared similar, we noted a stark reduction in expression patterns of *isl1a* in *foxg1a^a266^* larvae at 5 dpf when compared to heterozygous siblings (Fig. 6A,A′). Dorsoventral and anteroposterior support cells were labeled with *sost* and *tnfs10l3*, respectively, with *sost* marking regions important for hair cell regeneration (Lush et al., 2019; Thomas and Raible, 2019). Expression of *sost* and *tnsf10l3* was similar in control and *foxg1a^a266^* neuromasts (Fig. 6E-F′) recapitulating our earlier data of cell counts of *sost:nlsEos-*positive cells and HCR FISH of *tnfsf10l3* (Fig. 5E-H). We next looked for changes in genes linked to hair cell development and regeneration, specifically in the Wnt and Notch/Delta signaling pathways (Megerson et al., 2024; Romero-Carvajal et al., 2015). We noted that expression patterns for Notch/Delta ligands and markers *notch3*, *deltaD*, *her2*, *her4.1*, and *lfng* appeared similar in *foxg1a^a266^* larvae compared to controls (Fig. 6G-K′). We also see no obvious expression differences when comparing expression of the hair cell fate marker *atoh1a* in *foxg1a^a266^* larvae to controls (Fig. 6L,L′). Similarly, when we looked at expression of different Wnt ligands and β-catenin in *foxg1a^a266^* larvae we observe no obvious differences as compared to heterozygous controls (Fig. 5M-O′). Together, these expression profiles suggest that *isl1a* expression is regulated by Foxg1a function in posterior lateral line neuromasts.

### α-Isl1-labeled cells are reduced in *foxg1a^a266^* mutants during development

**Fig. 7.**
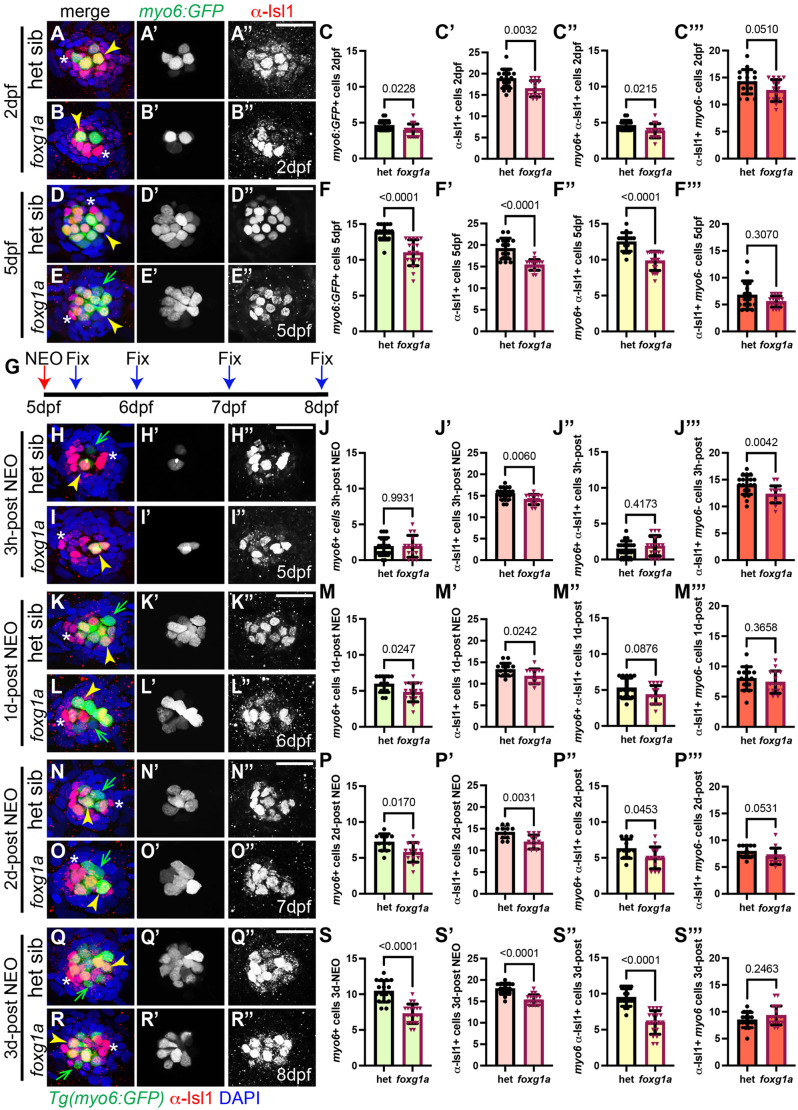
**α-Islet1 antibody-labeled cells are reduced in *foxg1a^a266^* mutants during development and regeneration.** Confocal projections of neuromasts showing hair cells labeled with *myo6:GFP* (green), central cells and hair cells labeled with α-Isl1 antibody (red), and nuclei labeled with DAPI (blue). Examples of hair cells that are both *myo6:GFP+* and α-Isl1+ are marked with yellow arrowheads, hair cells that are *myo6:GFP+* and α-Isl1- are marked by green arrows, and cells that are α-Isl1+ and *myo6:GFP*- are marked with white asterisks. (A-B″) Heterozygous sibling and *foxg1a^a266^* mutants neuromasts at 2 dpf. (C-C′″) Quantification of *myo6:GFP+* hair cells, α-Isl1+ cells, cells labeled with both *myo6:GFP* and α-Isl1, and cells labeled with only α-Isl1, *n*=17 NMs (nine embryos) heterozygous siblings and 15 NMs (eight embryos) *foxg1a^a266^* mutants. (D-E″) Heterozygous sibling and *foxg1a^a266^* mutant neuromasts at 5 dpf. (F-F′″) Quantification of *myo6:GFP+* hair cells, α-Isl1+ cells, cells labeled with both *myo6:GFP* and α-Isl1, and cells labeled with only α-Isl1, *n*=16 neuromasts (eight larvae) heterozygous siblings and 18 neuromasts (nine larvae) *foxg1a^a266^* mutants. (G) Timeline of NEO exposure at 5 dpf and fixation at 3 h-post NEO (5 dpf), 1 day-post NEO (6 dpf), 2 days-post NEO (7 dpf), and complete regeneration (8 dpf). (H-I″) Heterozygous sibling and *foxg1a^a266^* mutants neuromasts at 3 h-post NEO (5 dpf). (J-J′″) Quantification of *myo6:GFP+* hair cells, α-Isl1+ cells, cells labeled with both *myo6:GFP* and α-Isl1, and cells labeled with only α-Isl1, *n*=20 neuromasts (ten larvae) heterozygous siblings and 15 neuromasts (eight larvae) *foxg1a^a266^* mutants. (K-L″) Heterozygous sibling and *foxg1a^a266^* mutant neuromasts at 1 day-post NEO (6 dpf). (M-M′″) Quantification of *myo6:GFP+* hair cells, α-Isl1+ cells, cells labeled with both *myo6:GFP* and α-Isl1, and cells labeled with only α-Isl1, *n*=14 neuromasts (seven larvae) heterozygous siblings and 15 neuromasts (eight larvae) *foxg1a^a266^* mutants. (N-O″) Heterozygous sibling and *foxg1a^a266^* mutants neuromasts at 2 days-post NEO (7 dpf). (P-P′″) Quantification of *myo6:GFP+* hair cells, α-Isl1+ cells, cells labeled with both *myo6:GFP* and α-Isl1, and cells labeled with only α-Isl1, *n*=11 neuromasts (seven larvae) heterozygous siblings and 13 neuromasts (eight larvae) *foxg1a^a266^* mutants. (Q-R″) Heterozygous sibling and *foxg1a^a266^* mutants neuromasts at complete regeneration NEO (8 dpf). (S-S′″) Quantification of *myo6:GFP+* hair cells, α-Isl1+ cells, cells labeled with both *myo6:GFP* and α-Isl1, and cells labeled with only α-Isl1, *n*=17 neuromasts (nine larvae) heterozygous siblings and 19 neuromasts (nine larvae) *foxg1a^a266^* mutants. All data presented at mean±s.d., Mann–Whitney *U*-test. Scale bars: 20 µm.

The transcription factor *islet1a* (*isl1a*) is expressed in central support cells and is predicted to be in hair cell progenitor cells within zebrafish posterior lateral line neuromasts (Baek et al., 2022). The reduced expression of *isl1a* in 5 dpf *foxg1a^a266^* mutants we observed (Fig. 6A-A′) lead us to ask if we would see a concomitant reduction in the number of Isl1+ cells in the *foxg1a^a266^* mutant neuromast. Using an α-Isl1 antibody, we first looked at the migrating pLLP, where the first hair cells begin to form (Itoh and Chitnis, 2001). At 28 hpf, we found that a small number of nascent *myo6:GFP*+ hair cells in both mutant and control embryos which were not significantly different when comparing heterozygous control and *foxg1a^a266^* mutant primordia (Fig. S3D-E′,F). All the *myo6:GFP+* cells were also labeled with α-Isl1 (Fig. S3F″), though overall, there was a significant reduction in total α-Isl1-labeled cells in *foxg1a^a266^* mutants as compared to controls (Fig. S3D-E″,F′). We next examined α-Isl1 antibody labeling in maturing pLL neuromasts and observed that at 2 dpf and 5 dpf *foxg1a^a266^* fish exhibit reduced numbers of *myo6:GFP*+ hair cells and α-Isl1+ cells (Fig. 7A-E″,C,C′,F,F′). We also note there are significantly fewer *myo6:GFP+/*α-Isl1+ co-labeled cells at both the 2 dpf and 5 dpf time points in *foxg1a^a266^* mutants as compared to controls (Fig. 7A,-E″,C″,F″). Interestingly, when we quantified α-Isl1+, but *myo6:GFP-* cells, we noted that while there were fewer on average, there was no significant difference in mutant neuromasts as compared to heterozygous siblings at either the 2 dpf, or 5 dpf time points (Fig. 7A-E″,C′″,F′″). These data indicate that the reduced complement of α-Isl1+ cells may correspond to the reduced *myo6:GFP+* hair cell population.

### *foxg1a^a266^* neuromasts have fewer Isl1-labeled regenerating hair cells

We next sought to determine which cells are labeled by α-Isl1 antibody in the regenerating neuromast and if they are altered in *foxg1a^a266^* mutants. To assess this we fixed and labeled mutant and control fish expressing *myo6:GFP* with α-Isl1antibody at multiple time points during regeneration to better separate these populations (Fig. 7G). We first looked at α-Isl1 labeling 3 h-post NEO when most hair cells have been ablated, but new hair cells have not regenerated. At this time point we observed that there was still a large population of α-Isl1+ cells in both the heterozygous siblings and *foxg1a^a266^* mutants 3 h-post NEO (Fig. 7H-I″,J′) indicating that there is a subset of α-Isl1+ cells distinct from hair cells, and that there are significantly fewer α-Isl1+ cells in *foxg1a^a266^* larvae as compared to controls (Fig. 7J′-J″). As regeneration progresses, we see that *foxg1a^a266^* larvae form fewer hair cells per neuromast as compared to heterozygous siblings at 1 day-post NEO (Fig. 7K,K′,L,L′,M), 2 days-post NEO (Fig. 7N,N′,O,O′,P), and 3 days-post NEO (Fig. 7Q,Q′,R,R′,S). During these regeneration periods the total number of α-Isl1a+ cells in *foxg1a^a266^* larvae are significantly reduced compared to heterozygous controls (Fig. 7H-R”,J′,M′,P′,S’) at each time point. Fewer α-Isl1+/*myo6:GFP+* co-labeled regenerated hair cells are present in *foxg1a^a266^* larvae 2 days-post NEO (Fig. 7P″) and 3 days-post NEO (Fig. 7S″), whereas the α-Isl1+ non-hair cell population only exhibits a significant difference in mutants at 3 h-post NEO as compared to controls (Fig. 7J″′). No significant difference in the number of α-Isl1+ non-hair cells is observed at 1 day-post NEO (Fig. 7M″′), 2 days-post NEO (Fig. 7P′″), or 3 days-post NEO (Fig. 7S′″). These data indicate that α-Isl1+ labels a subset of neuromast cells, and that some of those α-Isl1+ cells include *myo6:GFP* hair cells. The hair cells which are also α-Isl1+ cells appear to be sensitive to Foxg1a function.

### α-Isl1+ cells represent a distinct population from *sost:nlsEos**+*** cells

**Fig. 8.**
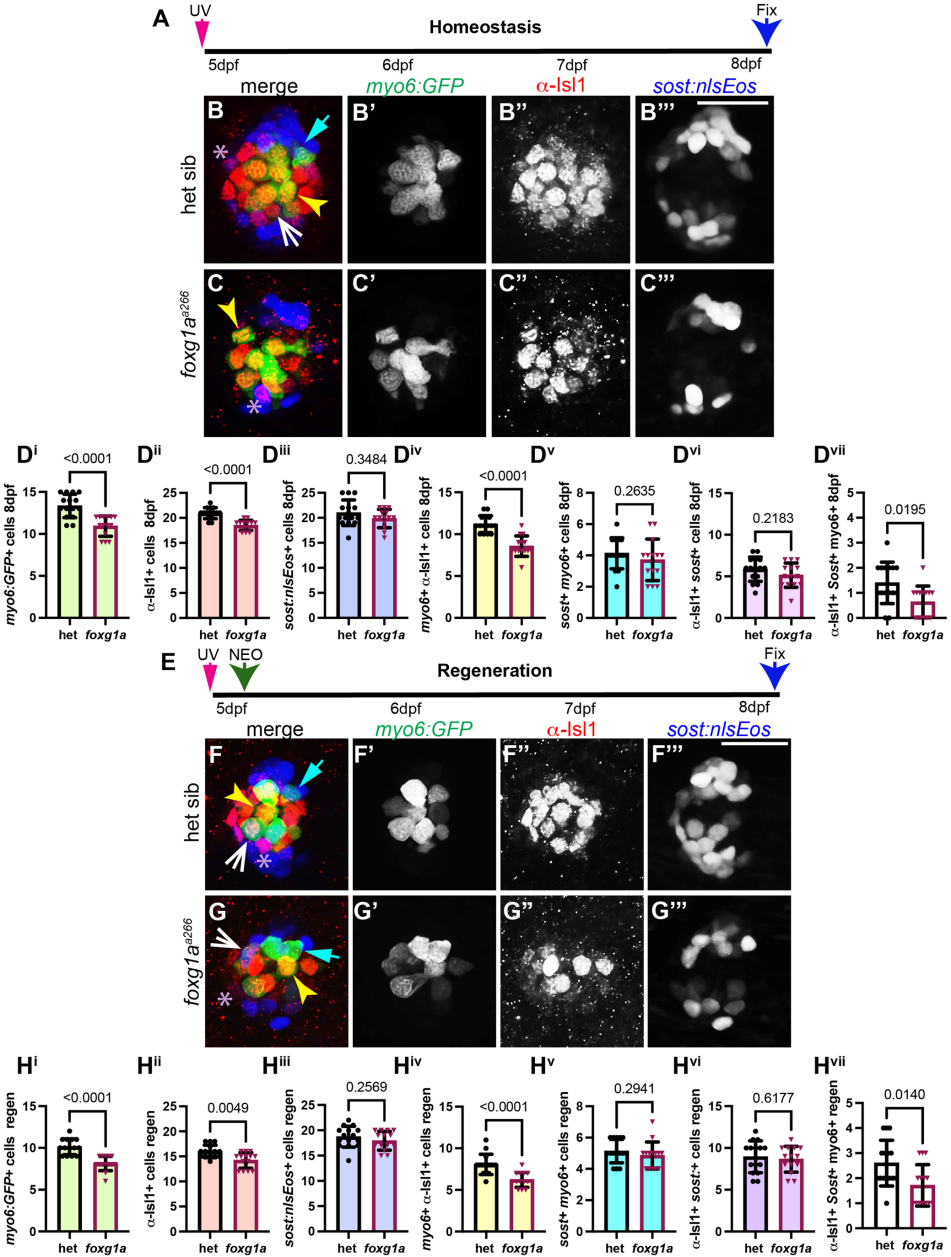
**α-Islet1 antibody and *sost:nlsEos* label differentially label regenerating hair cells.** Confocal projections of neuromasts showing hair cells labeled with *myo6:GFP* (green), central cells and hair cells labeled with α-Isl1 antibody (red), and *sost:nlsEos* cells (blue). Examples of hair cells that are both *myo6:GFP+* and *sost:nlsEos* are marked with cyan arrows, hair cells that are both *myo6:GFP+* and α-Isl1+ are marked with yellow arrowheads, hair cells that are *myo6:GFP+*, α-Isl1+, and *sost:nlsEos*+ are marked by white arrows, and cells that are α-Isl1+ and *sost:nlsEos+* are marked with purple asterisks. (A) Timeline of UV photoconversion of *sost:nlsEos* cells at 5 dpf, homeostasis for 3 days, and fixation at 8 dpf. (B-C′″) Heterozygous sibling and *foxg1^a266^* mutants neuromasts at 8 dpf. (D^i^-D^vii^) Quantification of *myo6:GFP+* hair cells (D^i^), α-Isl1+ cells (D^ii^), *sost:nlsEos+* cells (D^iii^) cells labeled with both *myo6:GFP* and α-Isl1 (D^iv^), hair cells labeled with *myo6:GFP* and *sost:nlsEos* (D^v^), cells labeled with *sost:nlsEos* and α-Isl1 (D^vi^), and cells labeled with *myo6:GFP,* α-Isl1, and *sost:nlsEos* (D^vii^), *n*=15 neuromasts (eight larvae) heterozygous siblings and 14 neuromasts (eight larvae) *foxg1^a266^* mutants. (E) Timeline of UV photoconversion of *sost:nlsEos* cells and NEO exposure at 5 dpf, regeneration for 3 days, and fixation at 8 dpf. (F-G′″) Heterozygous sibling and *foxg1^a266^* mutants neuromasts at 8 dpf. (H^i^-H^vii^) Quantification of *myo6:GFP+* hair cells (H^i^), α-Isl1+ cells (H^ii^), *sost:nlsEos+* cells (H^iii^) cells labeled with both *myo6:GFP* and α-Isl1 (H^iv^), hair cells labeled with *myo6:GFP* and *sost:nlsEos* (H^v^), cells labeled with *sost:nlsEos* and α-Isl1 (H^vi^), and cells labeled with *myo6:GFP,* α-Isl1, and *sost:nlsEos* (H^vii^), *n*=15 neuromasts (eight larvae) heterozygous siblings and 14 neuromasts (nine larvae) *foxg1^a266^* mutants. All data presented at mean±s.d. Significance was determined with Mann–Whitney *U*-tests. Scale bars: 20 µm.

Genetic expression analyses using scRNA-sequencing and cell lineage experiments suggest that neuromast cells are made up of distinct sub-populations that can contribute to hair cell regeneration differently based on expression profile and location (Baek et al., 2022; Lush et al., 2019; Thomas and Raible, 2019). The work by Thomas et al. demonstrated that a dorsoventral population labeled with *sost:nlsEos+* will give rise to the majority of hair cells regenerated following hair cell ablation (Thomas and Raible, 2019). We questioned how much overlap there may be between the α-Isl1+ support cell, α-Isl1+ hair cell, and the dorsoventral *sost:nlsEos+* populations and the effect of Foxg1a function on these cells. Using lineage tracing we looked at homeostatic development and regeneration by photoconverting *sost:nlsEos* in fish co-expressing *myo6:GFP* at 5 dpf then fixing at 8 dpf and then labeling with α-Isl1 antibody (Fig. 8A,E). Fish used for regeneration were exposed to NEO immediately after photoconversion at 5 dpf to allow lineage tracing of how *sost:nlsEos+* cells contribute to hair cell regrowth (Fig. 8E). We observed a decrease in the number of *myo6:GFP+* hair cells, α-Isl1+ cells, and *myo6:GFP/*α-Isl1+ co-labeled cells under both homeostatic conditions (Fig. 8B-B″,C-C″,D^i^,D^ii^,D^iv^) and during regeneration (Fig. 8F-F″,G-G″,H^i^,H^ii^,H^iv^) in *foxg1a^a266^* mutant larvae as compared to heterozygous siblings. The number of *sost:nlsEos+* cells at 8 dpf under homeostatic conditions and following regeneration showed no significant difference in *foxg1a^a266^* larvae compared to controls (Fig. 8F′″,G,G′″,H^i^). We were surprised the see that under both homeostatic and regenerative conditions *sost:nlsEos* and α-Isl1 label distinct groups of cells in the neuromast (Fig. 8B-C′″,D^v^-D^vi^,F-G′″,H^v^-H^vi^). In particular, we find that only a small subset of *myo6:GFP*+ hair cells are co-labeled with α-Isl1 and *sost:nlsEos* during homeostasis, and the number is significantly lower in *foxg1a^a266^* mutants (Fig. 8D^vii^). Following regeneration, we find a small increase in hair cells co-labeled with α-Isl1 and *sost:nlsEos* (Fig. 8H^vii^), though this population is smaller than total regenerated hair cells (Fig. 8H^i^) with significantly fewer in *foxg1a^a266^* mutants as compared to heterozygous siblings (Fig. 8H^vii^). Together, these data suggest that within the neuromast there are distinct subpopulations of cells which are labeled by *sost:nlsEos*, α-Isl1. Foxg1a function appears to specifically regulate α-Isl1+ hair cells during development and regeneration.

## DISCUSSION

Foxg1 is a transcription factor that has largely been studied for its role in neural development and subsequent developmental defects accompanying mutations within its gene (Wong et al., 2019; Zhao et al., 2009). The zebrafish homolog *foxg1a* has been well studied in the context of forebrain development, but our study is the first to look specifically at lateral line development and regeneration (Raj et al., 2020; Toresson et al., 1998; Zhao et al., 2009). Though recent research suggests *foxg1* is required for the proper development of electroreceptors in the lateral line of paddlefish (Minarik et al., 2024). Our work demonstrates that *foxg1a* is necessary for the proper development and regeneration of the zebrafish posterior lateral line. Loss of Foxg1a function results in delayed posterior lateral line development, a reduction in the proliferation of neuromast cells, and fewer hair cells. During regeneration, Foxg1a is required for proliferation, formation of new hair cells, and the number of α-Isl1-positive cells in the neuromast. We find that α-Isl1-labeled hair cells are specifically reliant on Foxg1a function during development and regeneration in the zebrafish posterior lateral line.

### Foxg1a functions to promote proper development of mechanosensory systems

In studies of the mammalian inner ear, Foxg1 is demonstrated to be necessary for proper morphological formation, innervation, and sensory cell development (Ding et al., 2020; Hwang et al., 2009). Foxg1 knockdown in mouse embryos results in shortened cochlea, greater proportion of inner ear hair cells, and a reduction in inner ear hair cell innervation (Ding et al., 2020; Hwang et al., 2009; Pauley et al., 2006; Zhang et al., 2020). When we looked at early pLL development in *foxg1a^a266^* mutant zebrafish, we noted a reduced rate of migration of the posterior lateral line primordium and delayed neuromast formation, though we found recovery of neuromast numbers by 5 dpf. It is interesting to note that murine models investigating the knockout of Foxg1 demonstrated an increase in hair cells (Pauley et al., 2006), in contrast to the zebrafish lateral line where we see a decrease in hair cell development. In mice, the loss or conditional knockdown of Foxg1 results in significant disruption to innervation of hair cells and the cochlea (Pauley et al., 2006; Zhang et al., 2020). When we examined innervation of hair cells in the *foxg1a^a266^*, we found a similar pattern to controls, suggesting the axonal extension of the sensory neurons associated with pLL may not be regulated by Foxg1a. More work is needed to better distinguish the precise role of Foxg1 mechanosensory system development as there is evident conservation of function within the tissue though possibly differing effects between species.

### Foxg1a is necessary for proliferative development and regeneration of sensory tissue

Hair and support cells of the zebrafish lateral line develop and regenerate mostly through proliferation and differentiation of support cell progenitors (Coombs et al., 2014; Ghysen and Dambly-Chaudiere, 2007; Kniss et al., 2016; Ma and Raible, 2009; Thomas et al., 2015). As mammalian Foxg1 is implicated in proliferative development of nervous tissue we reasoned the zebrafish Foxg1a may also regulate proliferation in the lateral line (Akol et al., 2022; Wong et al., 2019). Our work shows that loss of Foxg1a function results in reduced proliferation both during development and regeneration of the zebrafish pLL without an increase in cell death. Our work suggests a conserved role for Foxg1 in different support cell's ability to generate new hair cells via proliferation (Akol et al., 2022; Ding et al., 2020; Hwang et al., 2009). The timing of Foxg1 function may also be important, as murine work with loss of function (Pauley et al., 2006) or conditional knockdown (Zhang et al., 2020) showed differing effects during different developmental time points. This is also important as regeneration in murine models is observed during a short window in early development, while zebrafish hair cells retain the capacity to regenerate through the life of the animal (Brignull et al., 2009; Matsui and Cotanche, 2004).

### Islet1 in the posterior lateral line neuromasts

Islet1 is a LIM-homeodomain transcription factor known to regulate development of nervous tissue, neural epithelia, and otic tissue (Liang et al., 2011; Sun et al., 2008). In mice its expression is noted in developing support cell populations and nascent hair cells, with its expression in hair cells fading away as differentiation continues (Radde-Gallwitz et al., 2004). Work by others has shown *isl1a* is expressed in the central support cells of zebrafish neuromasts (Lush et al., 2019). Our work shows that α-Isl1-labels a subset of hair cells and additionally that loss of Foxg1a results in a decrease in the numbers of this cell population. It is also interesting that the *isl1a* expressing cells appear to be distinct from the dorsoventral cells *sost:nlsEos* cells and represent a separate group of Foxg1a sensitive hair cells. These data indicate there is possibly a role for *isl1a* in support cell progression to a hair cell fate that is at least in part regulated by *foxg1a.* Future work is needed to address linage tracing of central support cells to confirm their contribution to regenerating hair cells and to demonstrate the precise function of Foxg1a in these α-Isl1+ cells, particularly the target genes regulated by Foxg1a transcriptional function. It would also be interesting to investigate other known cellular functions of Foxg1a like autophagy, regulation of ROS, and cell cycle.

### Concluding remarks

Our study reveals a novel function of Foxg1a in regulating cellular proliferation in a of neuromast cells during development and regeneration, and in regulating the proper number of mechanosensory hair cells. We believe this work provides greater insight into the cellular and molecular mechanisms driving vertebrate mechanosensory tissue development and may elucidate new opportunities in mammalian hair cell regeneration.

## METHODS AND MATERIALS

### Zebrafish lines and maintenance

The following *D. rerio* (zebrafish) lines were used: wild-type*AB (ZIRC; http://zebrafish.org), *foxg1a^a266^* (Thyme et al., 2019), *Tg(sost:nlsEos)^w215^* (Thomas and Raible, 2019), *Tg(myosin6b:GFP)*^w186^ (Thomas and Raible, 2019), *Tg(sfrp1a:nlsEos)^w217^* (Thomas and Raible, 2019), *Tg(prim:lyn2mCherry)* (Wang et al., 2018), *TgBAC(neurod:EGFP)^nl1^* (Obholzer et al., 2008), and *(−4.9sox10:EGFP)^ba2^* (Dutton et al., 2009). Zebrafish were maintained and staged according to standard protocols (Kimmel et al., 1995). Experiments reported in this study were conducted on larvae between 24 hpf, and 8 dpf. Larvae were kept in E3 embryo medium (14.97 mM NaCl, 500 μM KCL, 42 μM Na_2_HPO_4_, 150 μM KH_2_PO_4_, 1 mM CaCl_2_ dihydrate, 1 mM MgSO_4_, 0.714 mM NaHCO_3_, pH 7.2). For all experiments, larvae were treated with tricaine (Syndel) prior to fixation in 4% paraformaldehyde/PBS (Thermo Fisher Scientific). All research was performed in accordance with the McGraw laboratory protocol #45344 approved by the UMKC IACUC committee. In laboratory zebrafish lines, sexual determination and differentiation has been shown to occur at ∼25 dpf (Kossack and Draper, 2019), after the timepoints analyzed in this study.

### Wholemount RNA *in situ* hybridization

Wholemount RNA *in situ* hybridization (WISH) was carried out using established protocols (Thisse and Thisse, 2008), modified with a 5-min Proteinase K (Thermo Fisher Scientific) treatment to preserve neuromast integrity. The probes used were: *atoh1a* (Itoh and Chitnis, 2001), *notch3* (Itoh and Chitnis, 2001), *deltaD* (Haddon et al., 1998), *foxg1a*, *tnfsf10l3*, *sost*, *sfrp1a*, *lfng*, *gata2a*, *her4.1*, *her2*, *wnt2*, *wnt10a*, *ctnnb1*, *six1a*, *six1b* and *isl1a*. Antisense probes were generated using established protocols (Thisse and Thisse, 2008) or using a PCR-based protocol (Logel et al., 1992).

### HCR fluorescent RNA *in situ* hybridization

HCR FISH was carried out following the manufacturer's protocol (Molecular Instruments). The probes used were *foxg1a-*B2 (4 pmol) and *tnfsf10l3-*B2 (4 pmol), with the amplifier B2-647 (Molecular Instruments). Larvae were subsequently labeled with DAPI and mounted using Fluorescent Mounting Media (EMD Millipore) to prevent fading.

### Immunohistochemistry, FM1-43FX and DASPEI labeling

Wholemount immunolabeling was performed using established protocols (Ungos et al., 2003) for all antibodies except when noted. The primary antibodies used were: α-Otoferlin antibody (α-Oto; mouse monoclonal, 1:200, DSHB, University of Iowa), α-Sox 2 antibody (rabbit polyclonal, 1:100, Invitrogen), α-Islet1 antibody (α-Isl1; mouse monoclonal, 1:100, DSHB, University of Iowa), and α-BrdU antibody (mouse monoclonal, 1:100, BD Biosciences). For α-Isl1 antibody labeling, larvae were fixed for 1-h at room temperature in %PFA and then incubated in water overnight at 4°C. Larvae in α-Isl1antibody were incubated for 2 days at room temperature. Secondary antibodies used were as follows: goat α-rabbit Alexa-647 antibody (1:1000, Invitrogen), goat α-mouse Alexa-568 antibody (1:1000, Invitrogen), and goat α-mouse Alexa-647 antibody (1:1000, Invitrogen). Mature hair cells were labeled by a 1-min incubation in 3 µM FM1-43FX (Invitrogen; Owens et al., 2008). Nuclei were labeled with 30 mM DAPI (Thermo Fisher Scientific). Antibody block was made per protocol using 2% goat serum. Hair cells were visualized in live larvae using 2-(4-(dimethylamino)styryl)-N-ethylpyridinium iodide (DASPEI; Invitrogen) according to established protocols (Harris et al., 2003).

### TUNEL Labeling

TUNEL labeling was conducted using the Click-iT Plus TUNEL Assay Alexa Fluor 594 (Invitrogen, C10618). The kit protocol was adapted for larval zebrafish; larvae were fixed in 4% paraformaldehyde in PBS for 1 h at room temperature. Proteinase K digestion was done with 10 ug/mL proteinase K in PBS for 10 min. All wash steps were doubled to reduce background.

### Neomycin exposure and regeneration

For hair cell ablation, 5 dpf larvae were incubated in 400 µM neomycin (NEO, Millipore-Sigma) in embryo medium for 0.5 h and then washed three times in fresh embryo medium. In experiments analyzing complete regeneration, larvae were collected 3 days post-NEO exposure. Shorter time periods of regeneration were also conducted per experimental need. For positive control TUNEL labeling following neomycin exposure, larvae were collected 1 h post NEO. For RNA *in situ* hybridization, larvae were collected 28 hpf, 2 dpf, and 5 dpf; for regeneration, larvae were collected at 3 h post NEO, 1 day post NEO, or 3 days post NEO.

### BrdU incorporation

Cellular proliferation was analyzed using 5-bromo-2′-deoxyuridine (BrdU; Millipore Sigma). BrdU incorporation was carried out using established protocols (Harris et al., 2003; Laguerre et al., 2005); larvae were incubated in 10 mM BrdU for 24 h at set times during development or following NEO-induced ablation, larvae were exposed to BrdU immediately after NEO for 24 h and then transferred to fresh E3 for 2 days and fixed at 8 dpf.

### Photoconversion

For photoconversion experiments using *Tg(sost:nlsEos)^w215^* or *Tg(sfrp1a:nlsEos)^w217^* fish, 5 dpf larvae were placed in a shallow depression slide and exposed to 405 nm light for 20 s using a Zeiss Imager.D2 compound microscope and a 10x objective. For regeneration experiments, photoconversion was carried out prior to NEO exposure. For live imaging of *Tg(sost:nlsEos)^w215^* fish, larvae were anesthetized using tricaine and embedded in 1.2% low melt agarose/E3 embryo medium.

### Image collection

For imaging of RNA *in situ* hybridization and immunohistochemistry, processed larvae were placed in 50% glycerol/PBS and mounted on slides. For imaging of HCR in FISH, larvae were mounted on slides in Fluorescent Mounting Media (Calbiochem) and imaged within 3 days of processing to prevent signal loss. Images were collected using a Zeiss 510 Meta LSM confocal microscope with Zen 2009 software or a Nikon AXR resonant scanning confocal microscope with NIS-Elements software. Images were processed using Fiji software (Schindelin et al., 2012) and brightness and contrast were adjusted using Photoshop (Adobe).

### Quantification and statistical analysis

For quantification of neuromast cell numbers, we took the mean of individual neuromasts from multiple fish and conducted appropriate analyses comparing control and mutant samples. Neuromasts from L1-L4 were analyzed for each fish and cells were manually counted. All statistical analysis was carried out using GraphPad Prism 10 (GraphPad Prism version 10.0.0 for Mac, GraphPad Software, San Diego, California USA, www.graphpad.com). The data presented represents discrete variables so use of nonparametric tests determined. A Mann–Whitney U two-tailed nonparametric test was used for pair-wise comparisons, a Kruskal–Wallis test with Dunn's multiple comparison tests between multiple conditions, and a Fisher's exact test was used to compare condition across groups. Significance was set at *P*<0.05. All data are presented as±standard deviation (s.d.). A power analysis was conducted from initial samples of hair cell averages with an alpha of 0.05, and beta of 0.2, which established a minimum sample size of six per group for counts. Power calculated via ClinCal.com sample size calculator for two independent samples.

**Table:**
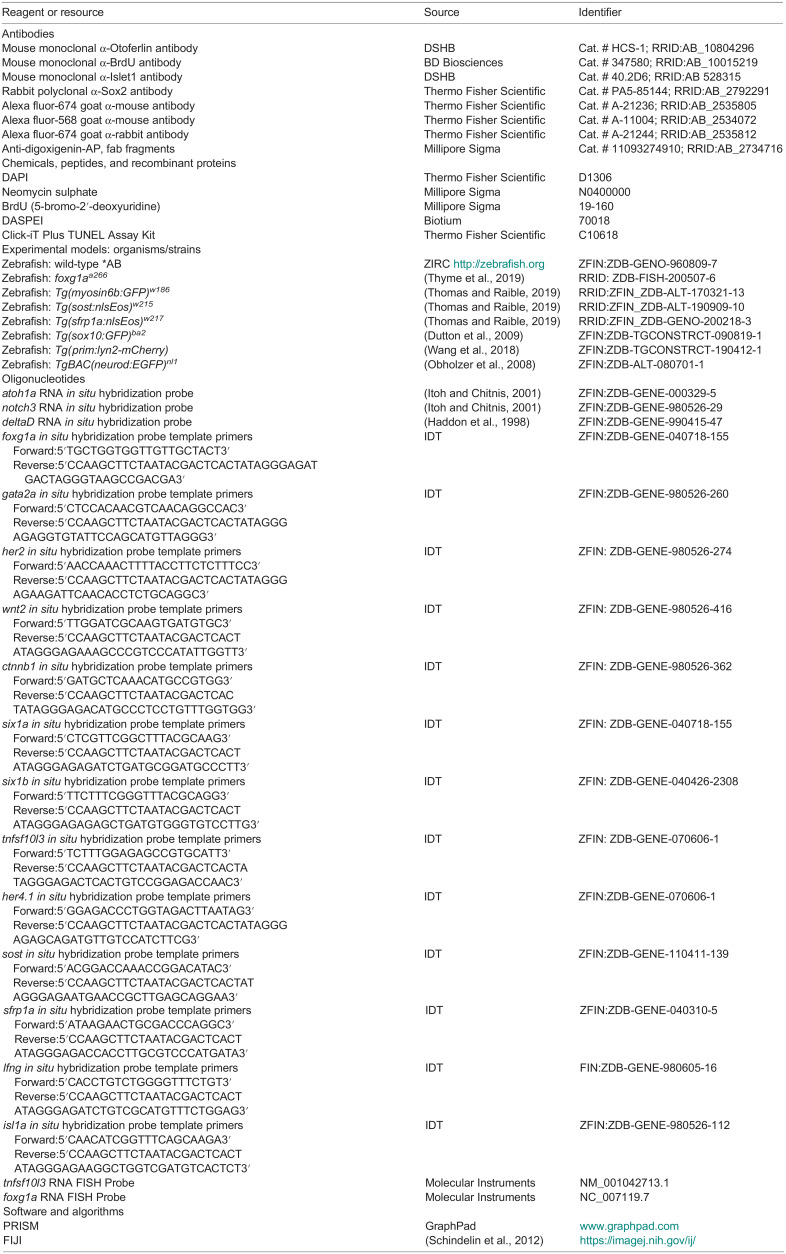


## Supplementary Material

10.1242/biolopen.060580_sup1 Supplementary information
