# Supplementary material for: foxg1a is required for hair cell development and regeneration in the zebrafish lateral line
Source: Biol Open. 2024 Sep 20;13(9):bio060580. doi: 10.1242/bio.060580 (PMC11423914; doi:10.1242/bio.060580)
Supplement: Supplementary information [file biolopen-13-060580-s1.pdf]

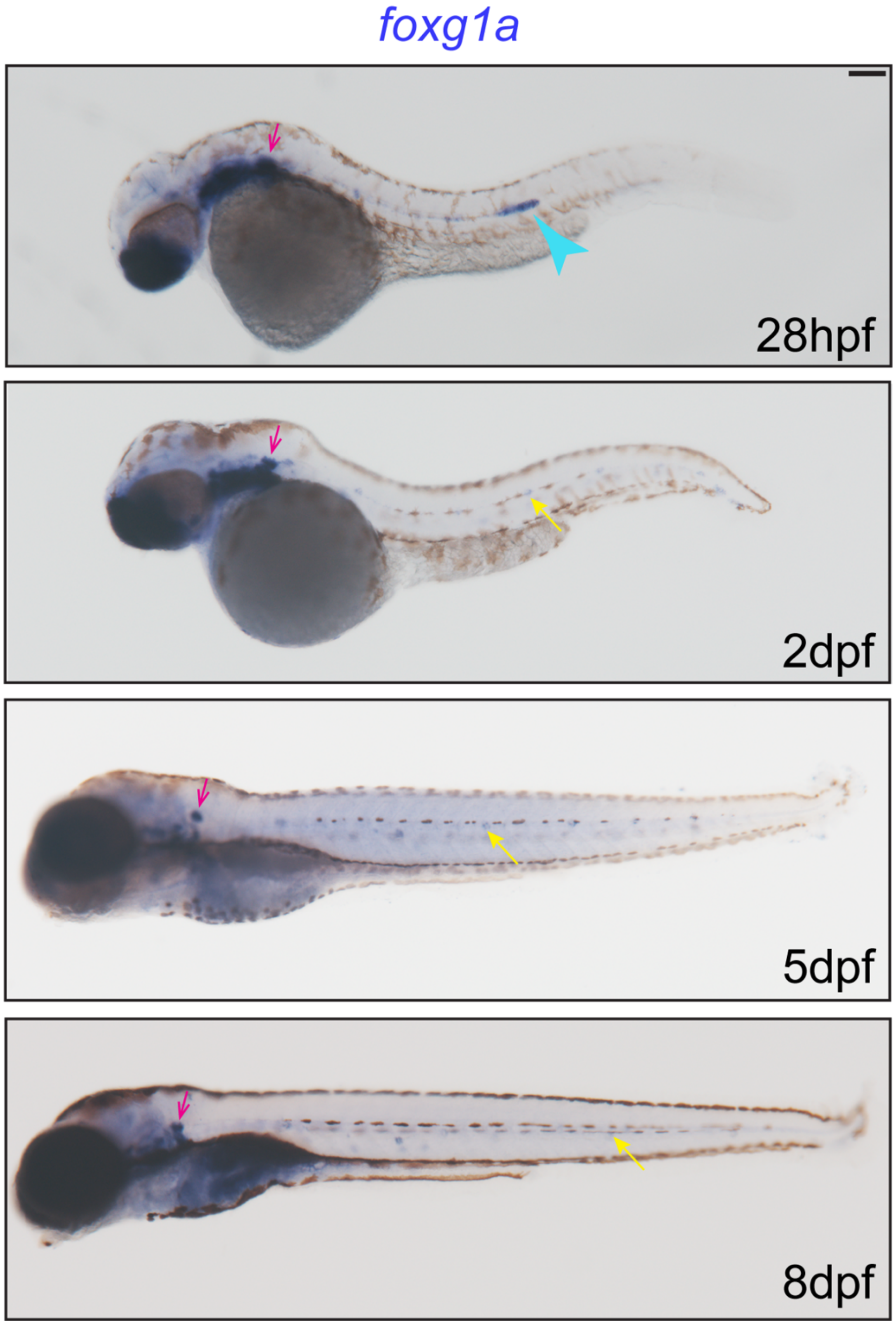

Fig. S1. Expression of *foxg1a* patterns in developing whole fish. DIC images of whole mount RNA in situ hybridization showing mRNA expression of *foxg1a* in AB wild-type fish at different developmental time points. (**A**) 28hpf embryo showing *foxg1a* expression in the forebrain, ear, eye, posterior lateral line ganglia (magenta arrow), and the posterior lateral line primordium (blue arrowhead). (**B**) 2dpf embryo showing *foxg1a* expression in the forebrain, ear, eye, posterior lateral line ganglia (magenta arrow), and neuromasts (representative neuromast indicated with a

Biology Open • Supplementary information

yellow arrow). (**C**) 5dpf embryo showing *foxg1a* expression in the ear, posterior lateral line ganglia (magenta arrow), and neuromasts (representative neuromast indicated with a yellow arrow). (**D**) 8dpf embryo showing *foxg1a* expression in the ear, posterior lateral line ganglia (magenta arrow), and neuromast (representative neuromast indicated with a yellow arrow). Scale bars = 100μm.

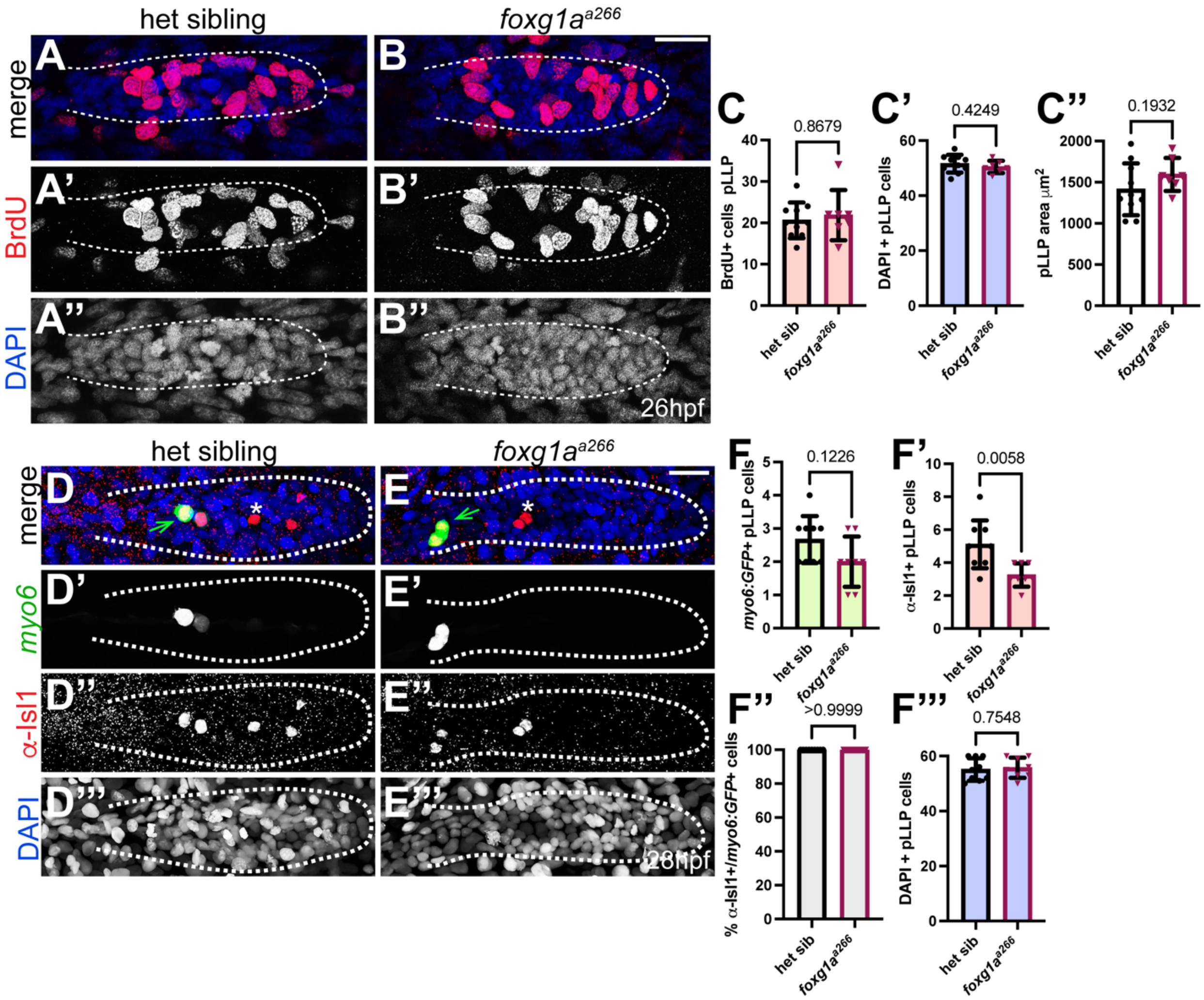

Fig. S2. Proliferation and early hair cell formation are normal in *foxg1a*^{a266} mutant embryos. (**A-B''**) Confocal projections of posterior lateral line primordium at 26hpf. Nuclei labeled with BrdU (red) and DAPI (blue) in heterozygous siblings (**A-A''**) and *foxg1a*^{a266} (**B-B''**) embryos. (**C**) Analysis of total BrdU labeled cells (**C'**) DAPI labeled cells, and (**C''**) primordia area in migrating primordium at 26hpf. n=10 heterozygous sibling, and n=7 *foxg1a.* (**D-E'''**) Confocal projections of posterior lateral line primordium at 28hpf. Hair cells are labeled with *myo6:GFP* (green), α-Isl1 labeled cells (red), and nuclei labeled with BrdU (blue), green arrows indicate myo6:GFP+/α-Isl1+ hair cells and yellow asterisks indicate α-Isl1+ cells. (**D',E'**) Heterozygous sibling and *foxg1a*^{a266} mutants pLLP hair cells labeled with *myo6:GFP*. (**D'',E''**) Heterozygous sibling and *foxg1a*^{a266} mutants pLLP α-Isl1 labeled cells. (**D''',E'''**) Heterozygous sibling

Biology Open • Supplementary information

and *foxg1a*^{a266} mutants pLLP DAPI labeled nuclei. (**F-F'**) Quantification of *myo6:GFP* and α-Isl1 labeled cells. (**F''**) Quantification of % α-Isl1+ hair cells labeled with *myo6:GFP.* (**F'''**) Quantification of total pLLP cells. n=9 heterozygous primordia, n=8 *foxg1a*^{a266} primordia. Data presented as mean ± SD, Mann-Whitney U test. Scale bars = 20μm.

Biology Open • Supplementary information

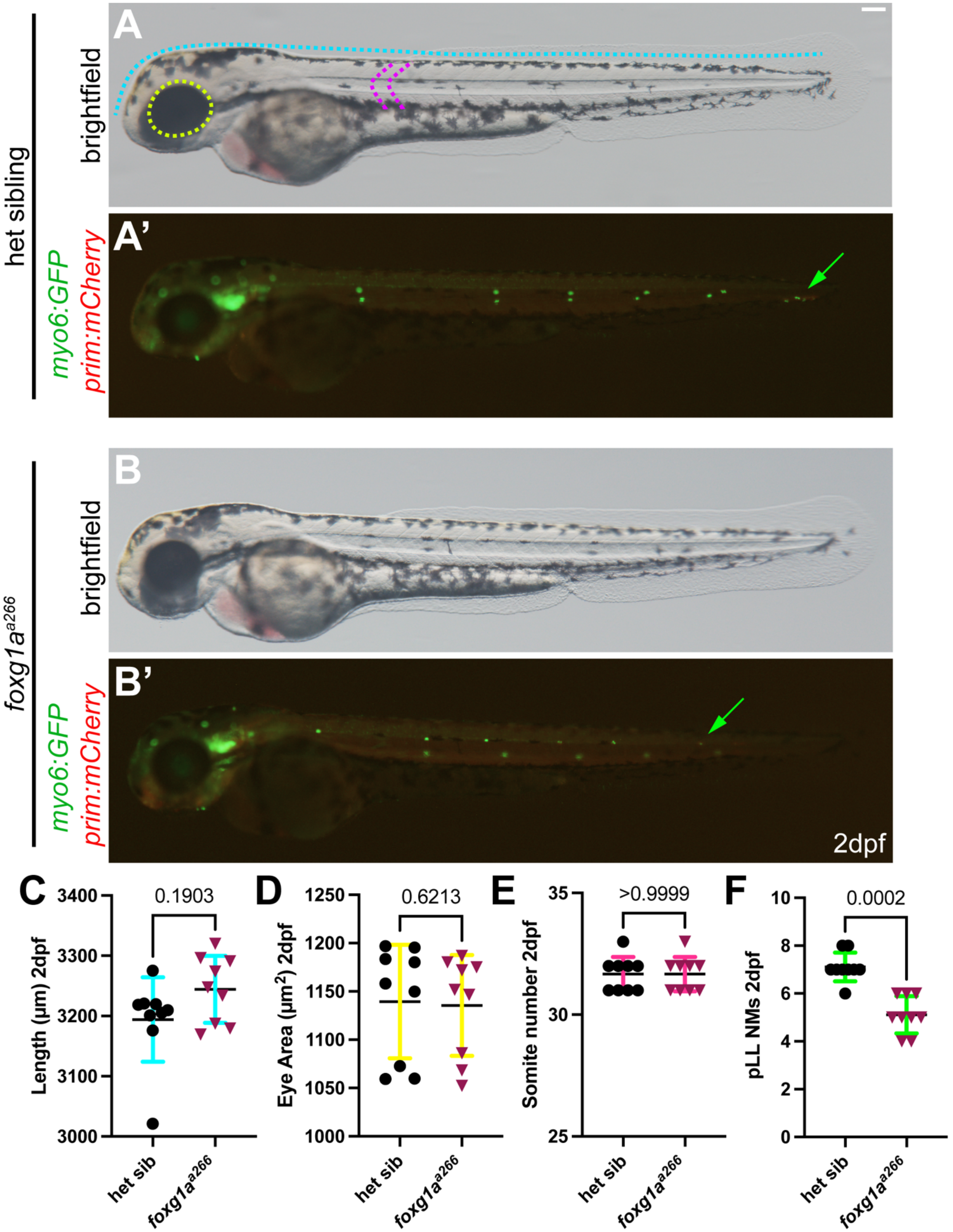

Biology Open • Supplementary information

Fig. S3. Body length, somite number, and eye area are unchanged in *foxg1a*^{a266} embryos. (**A-B'**) Brightfield and epifluorescent images of whole heterozygous sibling and *foxg1a*^{a266} mutant embryos at 2dpf. Hair cells labeled with *myo6:GFP* (green) and the lateral line is labeled with *prim:mCherry* (red). (**C**) Embryo length, as indicated by cyan dotted line, is measured in micrometers. (**D**) Embryo eye area, as indicated yellow dashed circle, is measured in square micrometers. (**E**) Quantification of embryo somite numbers; a representative somite is outlined by dashed magenta lines. (**F**) Quantification of neuromasts using *myo6:GFP* hair cells and *prim:mCherry* neuromasts. n=9 embryos for both conditions. Data presented as mean ± SD, Mann-Whitney U test. Scale bar = 50μm

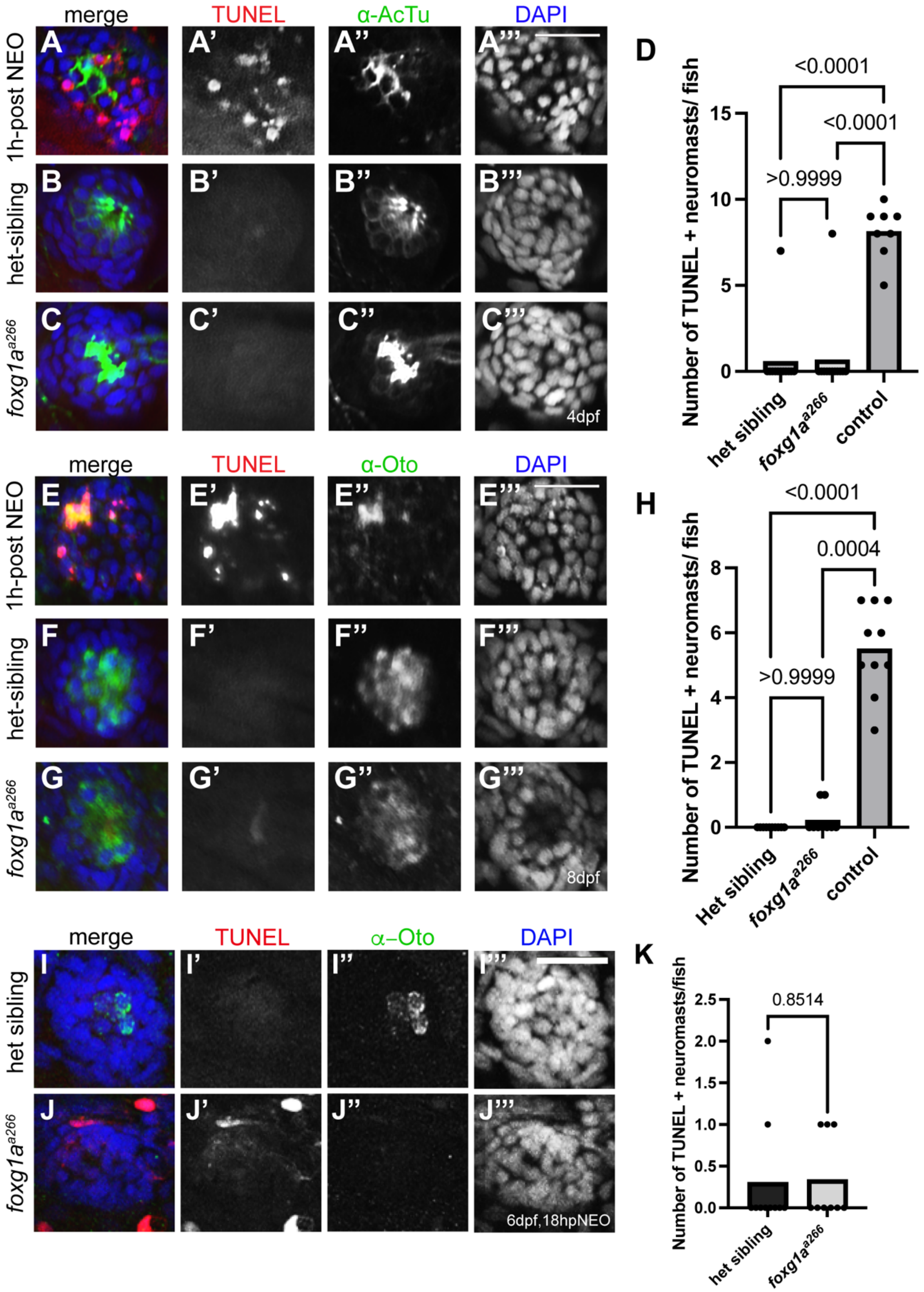

Biology Open • Supplementary information

Fig. S4. TUNEL labeling demonstrates no increase in apoptosis in *foxg1a^{a266}* mutants. (**A-C'''**) Confocal projections of NEO-exposed control, heterozygous sibling, and *foxg1a^{a266}* neuromasts 4dpf. (**C-C'''**) Hair cells marked with α-Acetylated Tubulin antibody (green), TUNEL-labeling (red) and nuclei labeled with DAPI (blue). (**D**) Quantification of the number of TUNEL positive neuromasts per fish. n=10 heterozygous siblings, n=9 *foxg1a^{a266}* mutants, n=10 NEO-exposed controls. (**E-G'''**) Confocal projections of NEO-exposed control, heterozygous sibling, and *foxg1a^{a266}* neuromasts 8dpf. Hair cells marked with α-Otoferlin antibody (green), TUNEL-labeling (red), and nuclei labeled with DAPI (blue). (**H**) Quantification of the number of TUNEL positive neuromasts per fish. n=12 heterozygous siblings, n=12 *foxg1a^{a266}* mutants, n=8 NEO-exposed controls. Data presented as mean ± SD, Kruskal-Wallis test. Scale bar =20μm. (**I-J'''**) Confocal projections of heterozygous sibling and *foxg1a^{a266}* mutant neuromasts 6dpf, 18 hour-post NEO. Hair cells marked with α-Otoferlin antibody (green), TUNEL-labeling (red), and nuclei labeled with DAPI (blue). (**K**) Quantification of the number of TUNEL positive neuromasts per fish. n=10 heterozygous siblings, n=9 *foxg1a^{a266}* mutants. Data presented as mean ± SD, Mann-Whitney U test. Scale bar =20μm.

Biology Open • Supplementary information

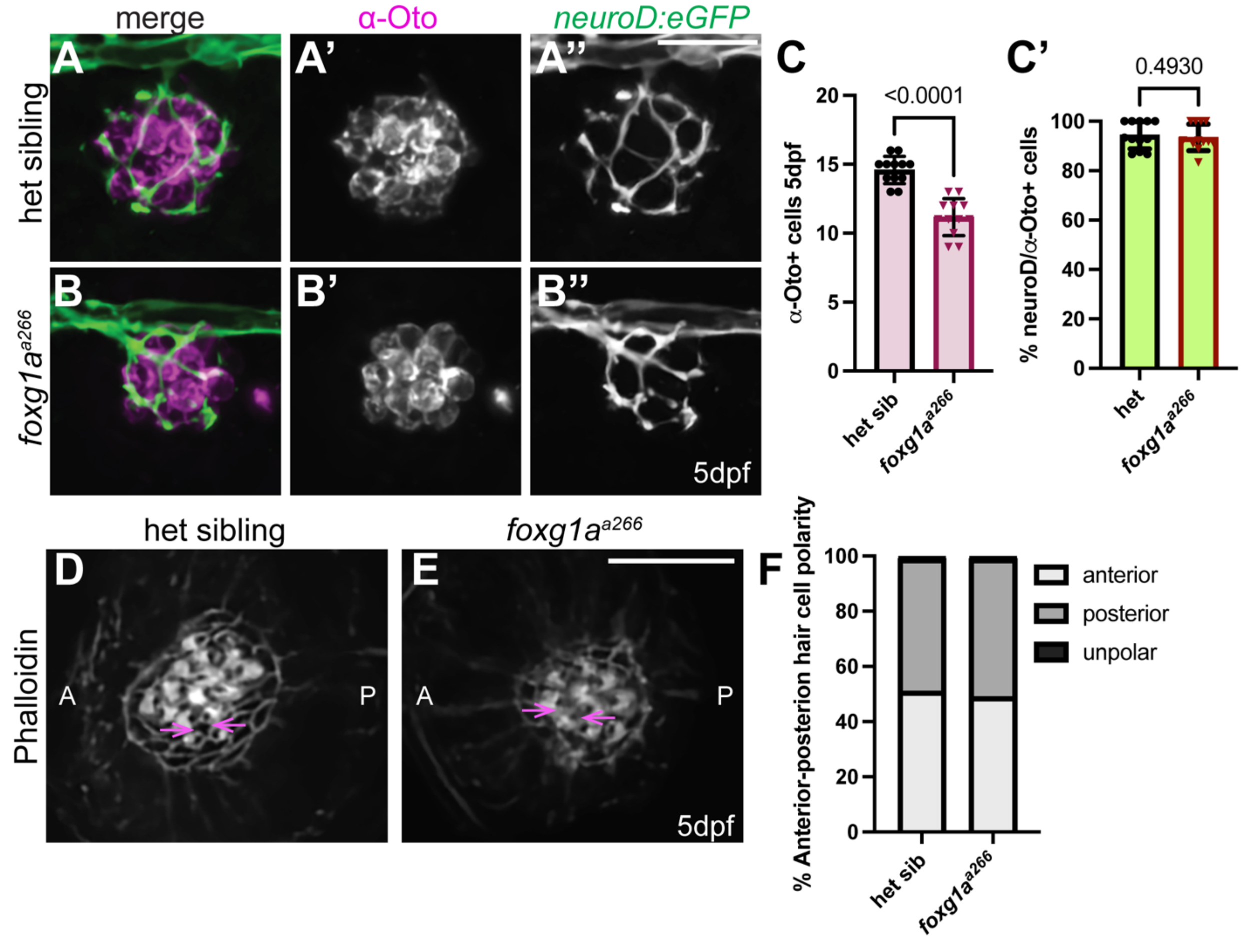

Fig. S5. Loss of Foxg1a function does not reduce innervation or change hair cell orientation. (**D-E''**) Confocal projections of 5dpf heterozygous sibling (**D-D''**) and *foxg1a*^{a266} (**E-E''**) showing hair cells labeled with α-Otoferlin (α-Oto; magenta), and axons expressing *TgBAC(neurod:EGFP)* (green). (**F**) Quantification of average number of α-Oto+ hair cells per neuromast and (**G**) quantification of hair cells surrounded by *TgBAC(neurod:EGFP*)+ neurons. n=12 neuromasts (6 larvae) per condition. Data presented as mean ± SD, Mann-Whitney U test. Scale bars = 20μm. (**H-I**) Confocal projects of phalloidin labeled cuticular plates in heterozygous sibling (**H**) and *foxg1a*^{a266} mutant (**I**) neuromasts, magenta arrows indicate representative examples of hair cell orientation. (**F**) Quantification of the percent anterior-posterior polarized or unpolarized hair cells at 5dpf. n=9 neuromasts (7 larvae) heterozygous sibling and n=12 neuromasts (8 larvae) *foxg1a*^{a266}. There is no significant difference in hair cell polarity, Fisher's exact test. Scale bar=10μm.

Biology Open • Supplementary information

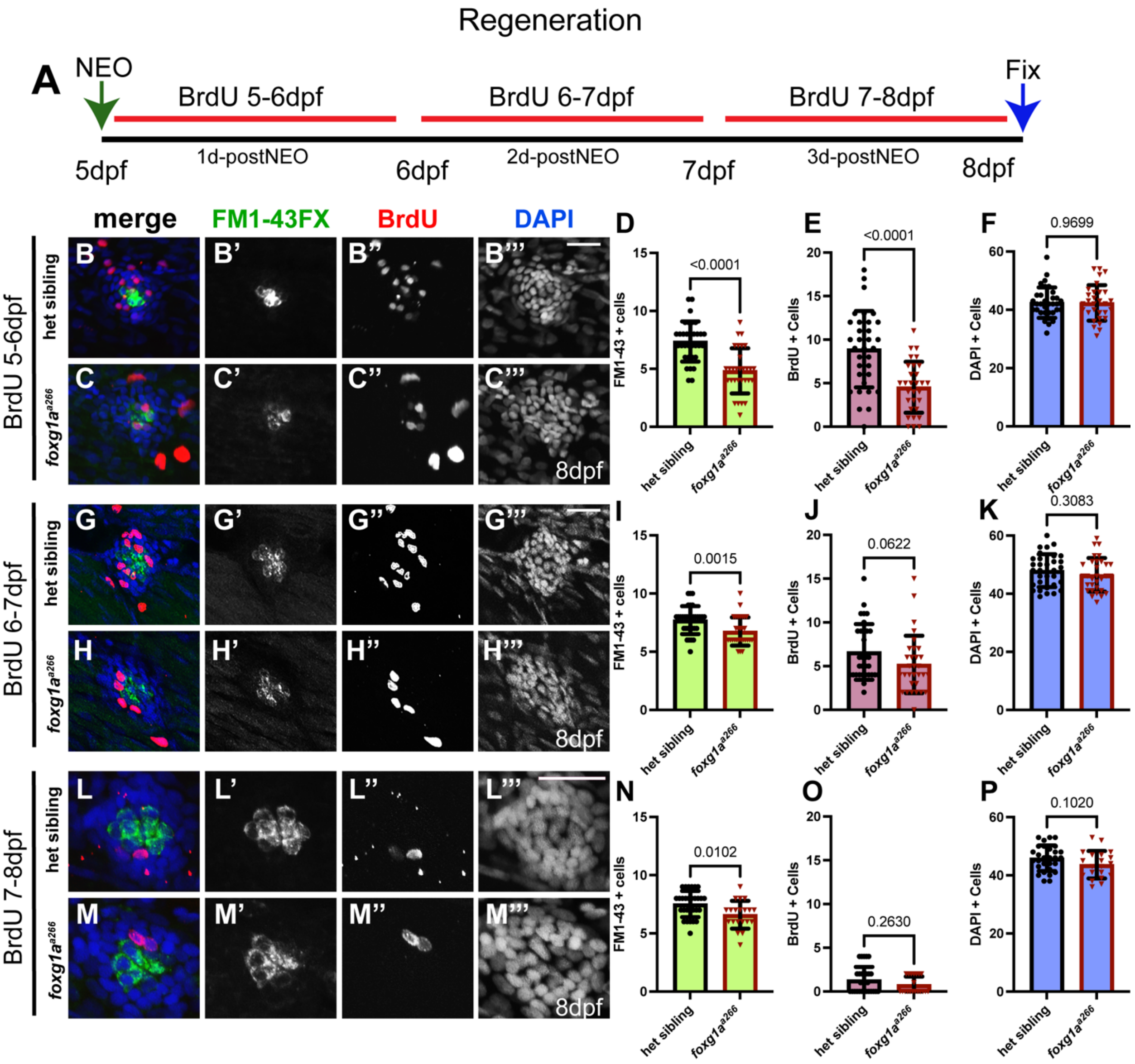

Fig. S6. The timing of proliferation during regeneration is not changed in *foxg1a^{a266}* mutant neuromasts. (**A**) Timeline of neomycin exposure at 5 dpf, followed by 24 h of BrdU incubation at 5-6 dpf, 6-7 dpf, or 7-8 dpf. Larvae were all fixed at 8 dpf. **(B-C''')** Confocal projections of 8 dpf heterozygous sibling and foxg1aa266 larvae exposed to neomycin sulfate followed by 24 hours of BrdU exposure, followed by 48 hours in embryo media. Hair cells stained with FM1-43FX (Green), with a-BrdU labeling (Red), and nuclei labeled with DAPI (Blue). **(D-F)**

Quantification of heterozygous siblings and *foxg1a*^{a266} mutant larvae hair cells (**D**), BrdU incorporation (**E**), and total nuclei (**F**). n= 33 neuromasts (7 larvae) heterozygous, n=30 neuromasts (7 larvae) *foxg1a*. (**G-H'''**) Confocal projections of 8dpf heterozygous sibling and *foxg1a*^{a266} larvae exposed to neomycin sulfate followed by 24 hours in embryo media, then 24 hours of BrdU exposure, and another 24 hours in embryo media. Hair cells stained with FM1-43FX (Green), α-BrdU labeling (Red), and nuclei labeled with DAPI (Blue). (**I-K**) Quantification of heterozygous siblings and *foxg1a*^{a266} mutant larvae hair cells (**I**), BrdU incorporation (**J**), and total nuclei (**K**). n=32 neuromasts (12 larvae) heterozygous, n=31 neuromasts (12 larvae) *foxg1a*. (**L-M'''**) Confocal projections of 8dpf heterozygous sibling and *foxg1a*^{a266} mutant larvae exposed to neomycin sulfate followed by 48 hours in embryo media then another 24 hours of BrdU exposure. Hair cells stained with FM1- 43FX (Green), α-BrdU labeling (Red) and nuclei labeled with DAPI (Blue). (**N-P**) Quantification of heterozygous siblings and *foxg1a*^{a266} mutant larvae hair cells (**N**), BrdU incorporation (**O**), and total nuclei (**P**). n=29 neuromasts (10 larvae) heterozygous larvae. n=20 neuromasts (8 larvae) *foxg1a*. All data presented as mean ± SD. Mann-Whitney U. Scale bar = 20μm.

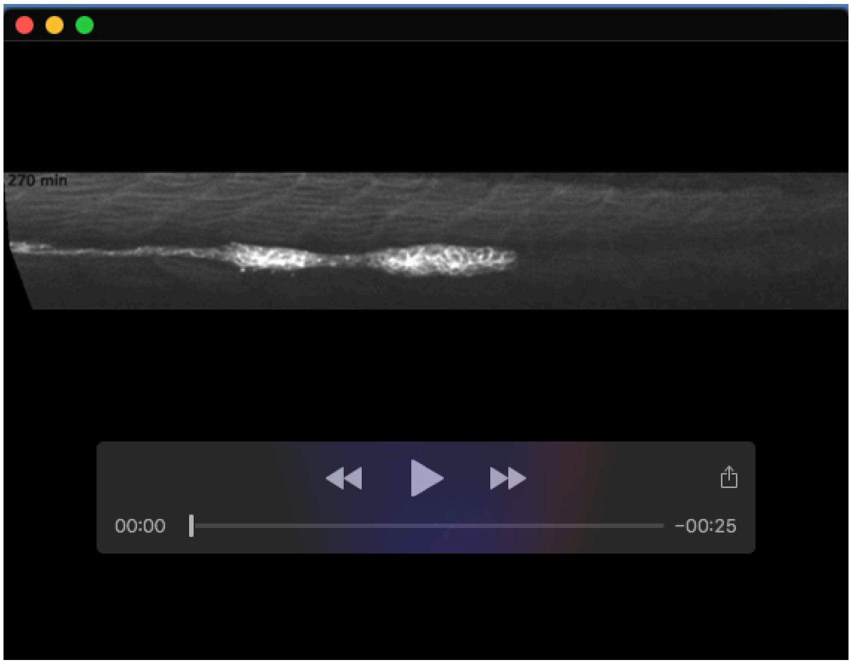

Movie 1. Time lapse live imaging of heterozygous posterior lateral line primordium migration from 33-48hpf at 18 minute intervals. Cell membranes of the primordium are labeled with *Tg(prim:lyn2mCherry)*. Embryos mounted in 1.2% low melt agarose. Imaging done on Zeiss 510 Meta confocal microscope using 20X objective. Scale bar = 20μm.

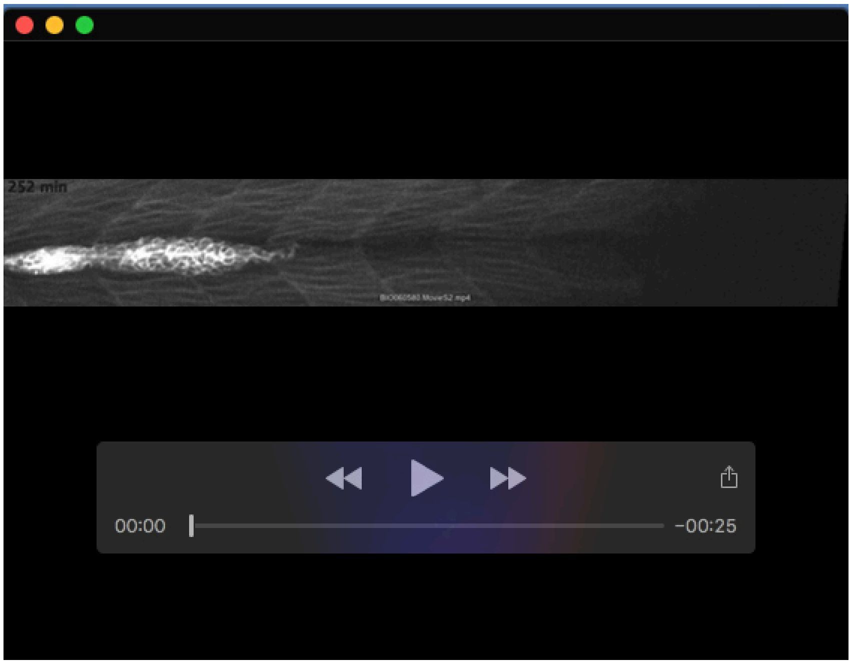

Movie 2. Time lapse live imaging of *foxg1a*^{a266} posterior lateral line primordium migration from 33- 48hpf at 18 minute intervals. Cell membranes of the primordium are labeled with *Tg(prim:lyn2mCherry)*. Embryos mounted in 1.2% low melt agarose. Imaging done on Zeiss 510 Meta confocal microscope using 20X objective. Scale bar = 20μm.

Biology Open • Supplementary information
